# Whole-Exome Sequencing Implicates the *USP34* rs777591A > G Intron Variant in Chronic Obstructive Pulmonary Disease in a Kashi Cohort

**DOI:** 10.3389/fcell.2021.792027

**Published:** 2022-02-07

**Authors:** Jingran Xu, Li Li, Jie Ren, Xuemei Zhong, Chengxin Xie, Aifang Zheng, Ayiguzali Abudukadier, Maimaitiaili Tuerxun, Sujie Zhang, Lifeng Tang, Dilare Hairoula, Xiaoguang Zou

**Affiliations:** ^1^ Department of Medical College, Shihezi University, Shihezi, China; ^2^ Department of Respiratory and Critical Care Medicine, First People’s Hospital of Kashi, Kashi, China

**Keywords:** whole-exome sequencing, HYDIN, USP34, rs12449210, rs777591, COPD

## Abstract

Genetic factors are important factors in chronic obstructive pulmonary disease (COPD) onset. Plenty of risk and new causative genes for COPD have been identified in patients of the Chinese Han population. In contrast, we know considerably little concerning the genetics in the Kashi COPD population (Uyghur). This study aims at clarifying the genetic maps regarding COPD susceptibility in Kashi (China). Whole-exome sequencing (WES) was used to analyze three Uyghur families with COPD in Kashi (eight patients and one healthy control). Sanger sequencing was also used to verify the WES results in 541 unrelated Uyghur COPD patients and 534 Uyghur healthy controls. WES showed 72 single nucleotide variants (SNVs), two deletions, and small insertions (InDels), 26 copy number variants (CNVs), and 34 structural variants (SVs), including g.71230620T > A (rs12449210T > A, NC_000,016.10) in the HYDIN axonemal central pair apparatus protein (*HYDIN*) gene and g.61190482A > G (rs777591A > G, NC_000002.12) in the ubiquitin-specific protease 34 (*USP34*) gene. After Sanger sequencing, we found that rs777591“AA” under different genetic models except for the dominant model (adjusted OR = 0.8559, 95%CI 0.6568–1.115, *p* > .05), could significantly reduce COPD risk, but rs12449210T > A was not related to COPD. In stratified analysis of smoking status, rs777591“AA” reduced COPD risk significantly among the nonsmoker group. Protein and mRNA expression of *USP34* in cigarette smoke extract-treated BEAS-2b cells increased significantly compared with those in the control group. Our findings associate the *USP34* rs777591“AA” genotype as a protector factor in COPD.

## Introduction

Chronic obstructive pulmonary disease (COPD) is a common and complicated disease of the lungs. It is characterized by continuous and irreversible airflow destruction due to chronic inflammation (Lozano et al., 2012). As of 2020, COPD is expected to become the fifth most prevalent burden of disease and the third leading cause of death worldwide (Vestbo et al., 2013).

Our epidemiological investigation shows the COPD prevalence in Kashi (Xinjiang, China) to be 17.01% (Li et al., 2021), which is higher than that in other parts of China. COPD development involves environmental factors (e.g., smoking, air pollution), genetic susceptibility, and infection (Zhang et al., 2014). A trend of familial aggregation in COPD patients is also documented (Wang et al., 2020). Scholars have found that some genes might be related to COPD genetic susceptibility of the Chinese Han population (Wang C. et al., 2018; Zhang et al., 2020). In contrast, the study for COPD is still lacking in Kashi.

Kashi city is located in the northwestern China, and more than 90% of the total local population in Kashi are Uyghurs (Zhang et al., 2018). The Uyghurs demonstrate a range of mixed Asian and European anthropological features (Xu and Jin 2008). At the same time, they usually do not marry other ethnic groups. Hence, their genomes are significantly different compared with the Han populations. Furthermore, the history, geographic location, and local customs of Kashi are quite different from other regions of China (Abuzhalihan et al., 2019; Abudureheman et al., 2021). These features make the Kashi Uyghurs a resourceful population for describing the ethnicity-specific variants associated with COPD.

The HYDIN axonemal central pair apparatus protein (*HYDIN*) gene is located on human chromosome 16 with a length of 423 kb (Laske et al., 2013). Population studies show that *HYDIN* mutations can cause primary ciliary dyskinesia (PCD) (Shapiro et al., 2018). PCD is a genetic disease with abnormal ciliary motility and is characterized by chronic respiratory infections (Olbrich et al., 2012; Kurkowiak et al., 2015). Respiratory infection is known to play an important role in the pathogenesis and progression of COPD (Leung et al., 2017).

The ubiquitin-specific protease 34 (*USP34*) gene is a member of the ubiquitin-specific protease family. *USPs* can regulate cell growth (Liu et al., 2019) and inhibit apoptosis (de Castro et al., 2019). Moreover, *USP34* regulates the Wnt pathway (Lui et al., 2011) and plays an important role in DNA damage (Sy et al., 2013). Studies confirm that *USP34* also has an influence in the NF-κB pathway (Truong et al., 2021). DNA damage, excessive apoptosis, and the NF-κB pathway are known to be associated with the development of COPD (Neofytou et al., 2012; Sauler et al., 2018; Canadas et al., 2020; Wang et al., 2021).

In the present study, eight people with COPD and one healthy person from three Uyghur families with COPD in Kashi were subjected to whole-exome sequencing (WES) to screen for the susceptibility genes and polymorphisms related to COPD. The two single nucleotide variants (SNVs) (rs12449210T > A in *HYDIN* and rs777591A > G in *USP34*) were determined to be studied because they were found for the first time in the Kashi COPD population. Furthermore, the association between the two SNVs and COPD risk have not been previously described. Based on the WES data sets, we continued to evaluate their relationship with clinical characteristics in a case-control study of 1075 people. Then, we hypothesized that the presence of SNVs combined with environmental factors (e.g., smoking) might regulate gene expression, thereby increasing the risk of COPD. Also, protein and mRNA expression of them were determined in cigarette smoke extract (CSE)-treated bronchial epithelial beas-2b (BEAS-2b) cells.

## Materials and Methods

### Collection of Information of COPD Families for WES

Eight COPD patients and one healthy control (HC) were selected for WES. All nine people were permanent residents of Kashi and aged >40 years. These Uyghur families were derived from a previous epidemiological investigation of COPD in Kashi. Families had ≥2 generations with COPD in three generations. The inclusion and exclusion criteria for the subjects were described by Gong et al. (2022).

Peripheral blood samples (5 ml) were obtained from each participant and transferred to EDTA-K_2_ Vacutainer tubes for DNA extraction. Apart from blood samples, the basic information of patients and HC were also collected: age, sex, body mass index (BMI), pulmonary function, smoking history, and other data. All pulmonary function tests were undertaken using a spirometer (Cosmed, Rome, Italy).

### Collection of Samples for a Case-Control Study

Recruited individuals were aged >40 years and from Kashi during 2018–2019. The study cohort was 541 Uyghur unrelated COPD patients and 534 Uyghur HCs from the First People’s Hospital of Kashi. For the COPD group, the inclusion criteria were people whose forced expiratory volume in the first second (FEV_1_)/forced vital capacity (FVC) < 0.70 after bronchodilator inhalation denotes airflow limitation. The exclusion criteria are identical to COPD family patients. For the HC group, the exclusion criteria were the same as HC of COPD family.

After providing written informed consent, all individuals were required to provide the same basic information as the family subjects. Similarly, 5 ml peripheral blood samples were obtained from each of the 1075 subjects for DNA extraction.

### Genetic Analyses

Genetic analyses, including WES, data analyses, variants selection, filtering strategy, Sanger sequencing, enrichment analyses, chemicals and reagents, Western blotting, and real-time RT-qPCR, had been described in the supplemental methods.

### Statistical Analyses

SPSS 18 (IBM, Armonk, NY, United States) and PLINK v1.07 (pngu.mgh.harvard.edu/∼purcell/plink/index.shtml) were employed for statistical analyses. Quantitative data are the mean ± SD or median (interquartile range). The independent *t*-test was used to compare the difference between age and BMI. We used the chi-square test to ascertain the influence of sex, smoking status, coal consumption, wood consumption, cigarettes per day, and cumulative quantity of active smoking. The difference in quitting smoking years was assessed by Fisher’s exact test. For parameters that did not have a normal distribution (e.g., annual household income, FEV_1_%, and FEV_1_/FVC), we used the Mann–Whitney U-test to evaluate differences. We tested for the Hardy–Weinberg equilibrium on each SNV among samples in the case-control study. Akaike’s information criteria (AIC) were used to calculate the genetic model for rs12449210 T > A and rs777591A > G (including genotype, dominant, recessive, allele, and additive). We used odds ratios (ORs) and corresponding 95% confidence intervals (CIs) by logistic regression analysis after correcting for sex, age, BMI, and smoking status to assess the relationship between SNVs and case–control groups. R function genpwr in package devtools was used to perform post hoc power estimation. The homogeneity test of stratified analyses was evaluated by the chi-square test. In the enrichment analyses, verification criteria of multiple SNVs were evaluated using the false discovery rate (FDR) with multiple corrections. The experimental data of Western blotting and real-time RT-qPCR were the mean ± SEM, and differences between groups were evaluated by one-way ANOVA. The replicates for the experiments were at least three times. *p* < .05 was considered significant.

## Results

### Family Members With COPD

#### WES

**FIGURE 1:**
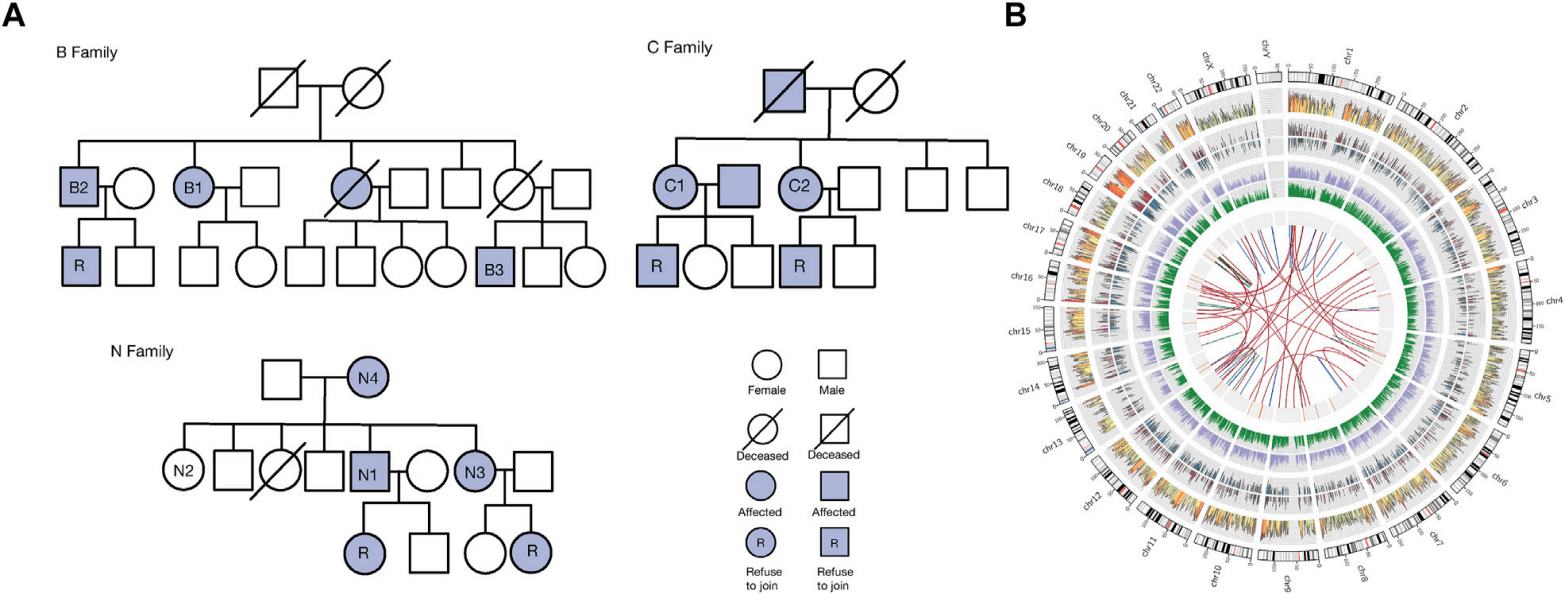
Family information and variants. **(A)**: Genealogical trees of three Uyghur families with COPD. Square = male, and circle = female. A square with a slash represents a dead male, and a circle with a slash denotes a dead female. A solid square represents a male with COPD, and the solid circle denotes a female with COPD. A solid square with “R” represents a COPD male who refused to join our study, and the solid circle with “R” denotes a COPD female who refused to join our study. **(B)**: Summary of all variations among exomes of family members with COPD. There are nine circles of information represented from the outside to the inside of this. Panel 1. Chromosome. 2. Map of SNVs. The density calculation is based on the number of SNVs in each window using log_10_. The color changes from red, yellow, and blue. 3. Density graph of inserts. The density calculation is based on the number of inserts in each window using log_10_. 4. Density of deletions. The density calculation is based on the number of deletions in each window using log_10_. 5. Density map of the variation sites in coding regions, including SNVs and InDels. The density calculation is based on the various sites in each window using log_10_. 6. Density map of the mutation sites in the noncoding area, including SNVs and InDels. The density calculation is based on the number of mutation sites in each window using log_10_. 7. Map of CNV locations. The area size represents the size of CNV. Red = gain, blue = loss (when the number of samples >1, there is no circle). 8. Map of SV position. The area size represents the size of SVs. Orange = DEL, green = INS. 9. Association of SV type diagram. Blue = INV, red = CTX, green = ITX.

WES was undertaken for Uyghur family members with COPD to identify common and significant variants. The genealogical trees of the three Uyghur families are described in Figure 1A. Eight patients and one healthy subject were included in Supplementary.Table S1. The family members with COPD were aged 48–90 (mean age = 63.63) and had always lived in Kashi.

After WES, there were ∼0.26 Tb of sequencing data. The minimum value of sequencing depth was 30×, the average sequencing depth was 116.29 ×. Q20 of all samples was >95%, Q30 was >90%, and the average coverage rate was >99.5%. We detected 100,193 SNVs, 11,009 deletions, and small insertions (InDels), 2915 copy number variants (CNVs), and 29,016 structural variants (SVs). To facilitate observation of all variants in family members with COPD, we used Circos to display all variants **(** Figure 1B **)**.

#### Analysis for SNVs

After WES, we obtained 100,193 SNVs. We undertook statistical analyses of all SNVs (Supplementary Figure S1). We found that the top three regions with the largest number of SNVs were the intron (43.47%), exon (42.52%), and noncoding RNA (5.76%) (Supplementary Figures S1A–C).

Then, 3096 SNVs were obtained after calculating the corresponding scores, SIFT and PolyPhen-2 (Supplementary Figure S1G). Finally, we obtained 72 SNVs after the filtering strategy (Supplementary Table S2).

#### Analysis for Small InDels, CNVs, and SVs

The top three regions with the largest number of InDels were the intron (71.90%), exon (8.28%), and noncoding RNA (8.28%) (Supplementary Figures S1D,E). Finally, we obtained 26 InDels (Supplementary Table S3).

After drawing a heat map for all 2915 CNVs, we detected the gain and loss of each chromosome (Supplementary Figure S2). Finally, we obtained two CNVs (Supplementary Table S4).

After analyzing the distribution of SVs in different regions of the genome, we found that the top three regions with the largest number of SVs were the intron (55.99%), exon (18.65%), and 3′ UTR (8.24%) (Supplementary Figure S1F). Finally, we obtained 34 SVs (Supplementary Table S5).

In conclusion, we obtained 72 SNVs, two CNVs, 26 InDels, and 34 SVs by the aforementioned filtering strategy.

#### Enrichment Analyses of Common Mutant Genes in Family Members with COPD

**FIGURE 2:**
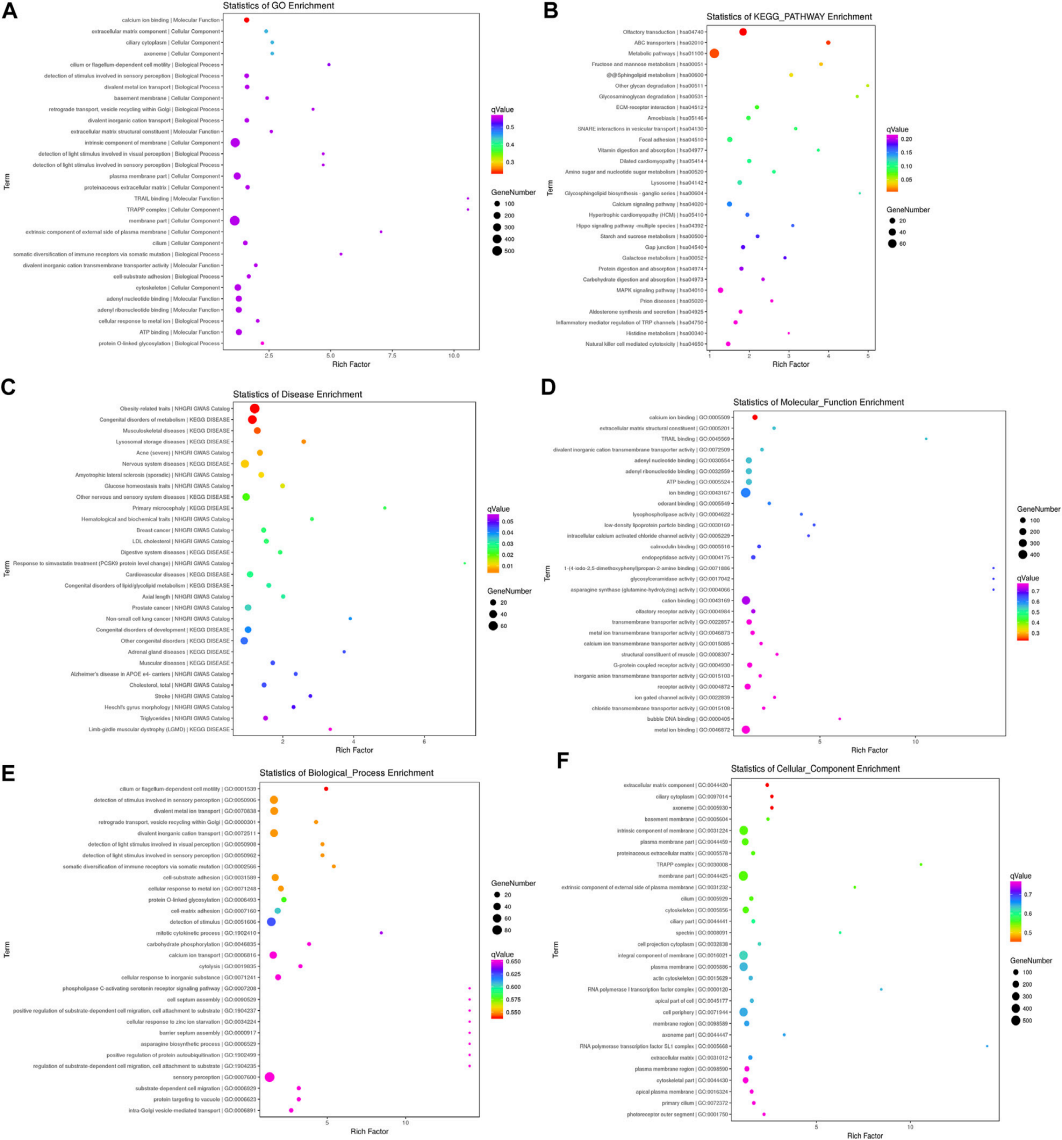
Bubble charts. **(A–C)**: The top 30 *q*-values for functional (GO database), pathway (KEGG database), and disease enrichment. **(D–F)**: The top 30 *q*-value GO terms among the categories of “cell component,” “molecular function,” and “biological process.” The abscissa “rich factor” represents the ratio of input or background frequency in the enrichment-analysis result. The bubble size represents the number of genes annotated to this functional entry for the mutant gene, and the color corresponds to the *q*-value in the enrichment-analysis result.

We selected the top 30 *q*-value GO terms, pathways, and diseases to draw a bubble chart (Figures 2A–C). We also selected the top 30 *q*-value GO terms among “cell component,” “molecular function,” and “biological process” to draw bubble charts (Figures 2D–F). The enrichment analyses indicate common mutant genes were related to cilium- or flagellum-dependent cell motility, ciliary cytoplasm, axoneme, calcium-ion binding, olfactory transduction, ABC transporters, obesity-related traits, and congenital disorders of metabolism.

### Case-Control Study

#### Clinical Characteristics of the COPD and Non-COPD Groups

A total of 1075 individuals were invited to participate (Table 1). The study cohort comprised 541 Uyghur COPD patients and 534 Uyghur HCs. Significant differences between COPD cases and HCs were observed with regard to age, sex, BMI, smoking status, FEV_1_%, and FEV_1_/FVC (*p* < .05 for all) but not for annual household income, coal consumption, wood consumption, cigarettes per day, cumulative quantity of active smoking, and quitting smoking years (*p* > .05 for all) (Supplementary Table S6).

**TABLE 1:** Clinical characteristics of the COPD and non-COPD groups.

Variables	Case *n* = 541 (%)	Control *n* = 534(%)	P
Age, years (mean ± SD)	61.11 ± 12.26	54.86 ± 10.73	<.001
Sex, n (%)			.009
Male	280 (51.75)	234 (43.82)	—
Female	261 (48.24)	300 (56.18)	—
BMI^a^ (kg/m^2^) (mean ± SD)	23.56 ± 4.22	25.55 ± 4.17	<.001
Annual household income (CNY) (median, range)	16,939 (10,294–23,442)	15,298 (9,762–23,057)	.197
Smoking status, n (%)			<.001
Never smoker	417 (77.08)	465 (87.08)	—
Former smoker	23 (4.25)	17 (3.18)	—
Current smoker	101 (18.67)	52 (9.74)	—
Coal consumption, n (%)			
Yes	513 (94.82)	516 (96.63)	.144
No	28 (5.18)	18 (3.37)	—
Wood consumption, n (%)			
Yes	519 (95.93)	500 (93.63)	.09
No	22 (4.07)	34 (6.37)	—
Pulmonary function (median, range)			
FEV_1_%	69.00 (53.92–82.00)	86.00 (74.00–98.00)	<.001
FEV_1_/FVC	0.62 (0.55–0.66)	0.80 (0.75–0.86)	<.001

a Body mass index.

FEV_1_. forced expiratory volume in 1 s; FVC, forced vital capacity.

#### Hardy–Weinberg Equilibrium of rs12449210T > A and rs777591A > G

Rs12449210T > A and rs777591A > G both met the criteria for the Hardy–Weinberg equilibrium (*p* > .05) (Table 2). Therefore, rs12449210T > A and rs777591A > G could be analyzed further.

**TABLE 2:** Analysis of the genotypes for rs12449210 of *HYDIN* and rs777591 of *USP34*.

SNV	HWE^a^	Model	Genotype	Case	Control	OR (95%CI)^b^	P^b^	OR (95%CI)^c^	P^c^
rs12449210 of HYDIN^d^	0.2933	Genotype	A/A	115 (21.58%)	113 (21.32%)	1.217 (0.867–1.709)	0.2563	1.132 (0.7821–1.639)	.5107
			T/A	270 (50.66%)	240 (45.28%)	1.345 (1.018–1.779)	0.03718	1.354 (0.9988–1.835)	.0509
			T/T	148 (27.77%)	177 (33.40%)	1	—	1	—
		Dominant	A/A,T/A	385 (72.23%)	353 (66.60%)	1.304 (1.004–1.695)	0.04664	1.281 (0.9632–1.704)	.08868
			T/T	148 (27.77%)	177 (33.40%)	1	—	1	—
		Recessive	A/A	115 (21.56%)	113 (21.32%)	1.015 (0.7575–1.361)	0.9193	0.9407 (0.6834–1.295)	.7079
			T/T,T/A	418 (78.42%)	417 (78.68%)	1	—	1	—
		Allele	A	500 (46.90%)	466 (43.96%)	1.126 (0.9492–1.336)	0.1732	1.089 (0.9042–1.311)	.3701
			T	566 (53.10%)	594 (53.04%)	1	—	1	—
		Additive	A/A	115 (21.56%)	113 (21.32%)	1.122 (0.9481–1.328)	0.1803	1.086 (0.9042–1.304)	.3774
			T/A	270 (50.66%)	240 (45.28%)	—	—	—	—
			T/T	148 (27.77%)	177 (33.40%)	—	—	—	—
rs777591 of USP34^e^	0.546	Genotype	A/A	51 (9.53%)	76 (14.23%)	0.5943 (0.3981–0.8871)	0.0109	0.5767 (0.3716–0.8948)	.01405
			G/A	248 (43.36%)	249 (46.63%)	0.882 (0.6828–1.139)	0.3367	0.9444 (0.7148–1.248)	.6872
			G/G	236 (44.11%)	209 (39.14%)	1	—	1	—
		Dominant	A/A,G/A	299 (55.89%)	325 (60.86%)	0.8147 (0.6386–1.039)	0.09923	0.8559 (0.6568–1.115)	.2497
			G/G	236 (44.11%)	209 (39.14%)	1	—	1	—
		Recessive	A/A	51 (9.53%)	76 (14.23%)	0.635 (0.4355–0.9259)	0.01826	0.5942 (0.3927–0.8993)	.01381
			G/G,G/A	484 (90.47%)	458 (85.77%)	1	—	1	—
		Allele	A	350 (32.71%)	401 (37.55%)	0.8086 (0.6768–0.966)	0.01923	0.8188 (0.6746–0.9938)	.04304
			G	720 (67.29%)	667 (62.45%)	1	—	1	–
		Additive	A/A	51 (9.53%)	76 (14.23%)	0.8046 (0.6717–0.9637)	0.01819	0.8146 (0.6693–0.9915)	.04085
			G/A	248 (43.36%)	249 (46.63%)	—	—	—	—
			G/G	236 (44.11%)	209 (39.14%)	—	—	—	—

a HWE: Hardy–Weinberg equilibrium for all subjects; OR, odds ratio; CI, confidence interval.

Statistic power (1-β): for rs12449210 and rs777591 are 52.24 and 84.38%.

b Logistic regression: Uncorrected for sex, age, smoking status, and BMI, *p* < .05 denotes significance.

c Logistic regression: Corrected for sex, age, smoking status, and BMI, *p* < .05 denotes significance.

d Rs12449210 of *HYDIN*: 533 COPD patients in case group, and 530 healthy people in control group.

e Rs777591 of *USP34*: 535 COPD patients in case group, and 534 healthy people in control group.

The call rate for rs12449210 of *HYDIN*, and rs777591 of *USP34* were 98.88% (1,063/1,075) and 99.44% (1,069/1,075), respectively.

#### Analysis of Genotypes of rs12449210T > A and rs777591A > G in the Case-Control Study

We undertook genetic model analysis (genotype, dominant, recessive, allele, and additive) on the SNVs of rs12449210T > A and rs777591A > G (Table 2). Because a few subjects might have *de novo* mutations (Francioli et al., 2015; Besenbacher et al., 2016; Garimella et al., 2020) in the primer binding region, the call rate of rs12449210 of *HYDIN* and rs777591 of *USP34* were 98.88% (1,063/1,075) and 99.44% (1,069/1,075).

For rs777591A > G located on *USP34*, after correcting for sex, age, smoking status, and BMI, this site significantly decreased the risk of COPD with the “AA” genotype based on the genotype model (OR: 0.5767, 95%CI: 0.3716–0.8948, *p* < .05) and recessive model (OR: 0.5942, 95%CI: 0.3927–0.8993, *p* < 0.05). In the allele model, the “A” genotype was related to a reduced risk of COPD (OR: 0.8188, 95%CI: 0.6746–0.9938, *p* < .05). For the additive model, a reduced tendency of COPD risk was also present (OR: 0.8146, 95%CI: 0.6693–0.9915, *p* < .05).

For rs12449210T > A located on *HYDIN* before correction “TA” or “AA + TA” increased the COPD risk under the genotype and dominant models (genotype model: OR: 1.345, 95%CI: 1.018–1.779, *p* < .05; dominant model: OR: 1.304, 95%CI: 1.004–1.695, *p* < .05). After correction, a significant effect was not present in the five models (*p* > .05).

#### Stratified Analysis of rs12449210T > A and rs777591A > G in the Case–Control Study

We undertook stratified analysis for the relationship between rs12449210T > A and rs777591A > G and COPD risk based on smoking status and FEV_1_%.

The beneficial effects of “AA” in rs777591A > G was more evident in nonsmokers in the genotype model [adjusted odds ratio (aOR): 0.511, 95%CI: 0.3102–0.8417], recessive model (aOR: 0.514, 95%CI: 0.3208–0.8237), allele model (aOR: 0.7978, 95%CI: 0.6415–0.9922), and additive model (aOR: 0.8053, 95%CI: 0.6507–0.9964). However, in the smoker group, the protective effects were not significant (Table 3).

**TABLE 3:** Analysis of genotypes for rs777591 of *USP34* among nonsmokers and smokers.

SNV	Model	Genotype	Nonsmokers				Smokers				Heterogeneity test	
			Case	Control	OR (95%CI)^a^	P^a^	Case	Control	OR (95%CI) ^a^	P^a^	χ^2^	P
rs777591 of USP34	Genotype	A/A	31 (8.33%)	67 (14.41%)	0.511 (0.3102–0.8417)	.0084	14 (11.38%)	9 (13.04%)	0.8972 (0.3212–2.506)	0.8359	0.93	.3340
		G/A	183 (49.19%)	217 (46.67%)	0.9889 (0.7282–1.343)	.9428	50 (40.65%)	32 (46.38%)	0.7609 (0.388–1.492)	0.4263	0.48	.487
		G/G	158 (42.48%)	181 (38.92%)	1	—	59 (47.97%)	28 (40.58%)	1	—	—	—
	Dominant	A/A, G/A	214 (57.53%)	284 (61.08%)	0.8723 (0.6514–1.168)	.3591	64 (52.03%)	41 (59.42%)	0.7887 (0.4178–1.489)	0.4642	0.08	.775
		G/G	158 (42.47%)	181 (38.92%)	1	—	59 (47.97%)	28 (40.58%)	1	—	—	—
	Recessive	A/A	31 (8.33%)	67 (14.41%)	0.514 (0.3208–0.8237)	.0057	14 (11.38%)	9 (13.04%)	1.026 (0.3883–2.709)	0.9592	1.57	.210
		G/G, G/A	341 (91.67%)	398 (85.59%)	1	—	109 (88.62%)	60 (86.96%)	1	—	—	—
	Allele	A	245 (32.93%)	351 (37.74%)	0.8053 (0.6507–0.9964)	.0463	78 (31.71%)	50 (36.23%)	0.8817 (0.5501–1.413)	0.601	0.12	.732
		G	499 (67.07%)	579 (62.26%)	1	—	168 (68.29%)	88 (63.77%)	1	—	—	—
	Additive	A/A	31 (8.33%)	67 (14.41%)	0.7978 (0.6415–0.9922)	.0423	14 (11.38%)	9 (13.04%)	0.8865 (0.5585–1.407)	0.6092	0.16	—
		G/A	183 (49.19%)	217 (46.67%)	—	—	50 (40.65%)	32 (46.38%)	—	—	—	.686
		G/G	158 (42.48%)	181 (38.92%)	—	—	59 (47.97%)	28 v40.58%)	—	—	—	—

a Logistic regression: Corrected for sex, age, and BMI, *p* < .05 denotes significance.

For rs12449210T > A (Supplementary Table S7), no differences were observed in smokers and nonsmokers before or after correction of potentially influencing factors.

Rs12449210T > A and rs777591A > G did not show an obvious difference in FEV_1_% (Supplementary Table S8).

#### Expression of USP34 and Iκbα in CSE-Treated BEAS-2b Cells

**FIGURE 3:**
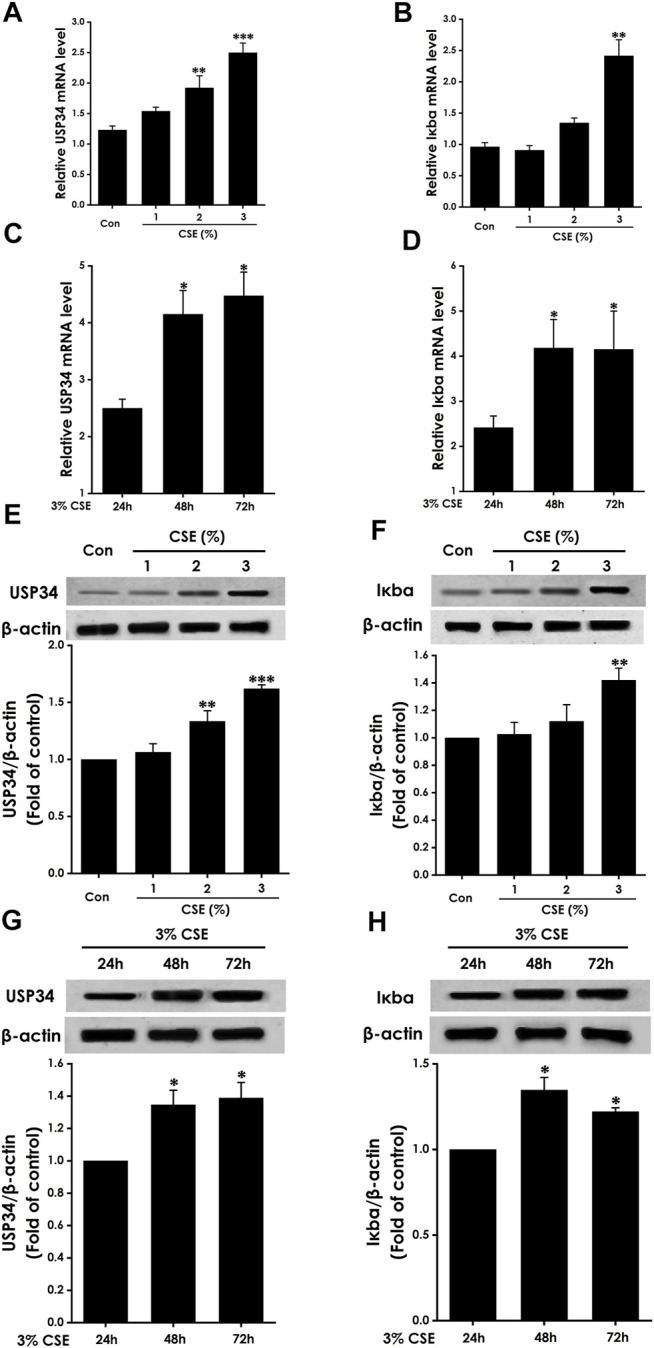
Expression of USP34 and Iκbα in CSE-treated BEAS-2b cells. mRNA expression assessed by RT-qPCR for USP34 **(A,C)** or Iκbα **(B,D)** after treatment with **(A,B)** 0%–3% CSE for 24 h (*n* = 3) or **(C,D)** 3% CSE for different times (*n* = 3). Protein expression assessed by Western blotting for USP34 **(E,G)** or Iκbα **(F,H)** after treatment with **(E,F)** 0%–3% CSE (*n* = 3) or **(G,H)** 3% CSE for different times (*n* = 3). Anti-β-actin antibody was used as a control in Western blotting. *p < .05, **p < .01, and ***p < .001, versus control or 24-h group.

We wished to determine the impact of CSE on expression of USP34 and Iκbα. We measured expression in BEAS-2b cells treated with 0%–3% CSE for 24 h. Compared with the control group, 3% CSE obviously increased expression of USP34 and Iκbα protein (Figures 3E,F) and mRNA (Figures 3A,B).

To determine the impact of different treatment times on expression of USP34 and Iκbα, we measured expression in BEAS-2b cells treated with 3% CSE for 24, 48, and 72 h. Compared with the 24 h group, treatment for 48 and 72 h significantly increased expression of USP34 and Iκbα protein (Figures 3G,H) and mRNA (Figures 3C,D).

## Discussion

A total of 72 SNVs, two CNVs, 26 InDels, and 34 SVs were obtained by WES from three Uyghur families with COPD. Two SNVs, rs12449210T > A of *HYDIN* and rs777591A > G of *USP34,* were chosen to verify in a large Uyghur population. We found that “AA” in rs777591A > G was a protective factor against COPD, whereas rs12449210T > A was not related to COPD susceptibility in Kashi COPD population. mRNA and protein expression of USP34 and Iκbα were obviously increased in CSE-treated BEAS-2b cells *in vitro* study.

WES has been used widely in the discovery of pathogenic genes (Adalsteinsson et al., 2017; Epi25 Collaborative, 2019; Petrovski et al., 2019). WES could be used to detect high-frequency, low-frequency, and even rare variants that have important roles in the occurrence of complex diseases and could be employed to discover new variants (Meienberg et al., 2015).

An approach, used in Mendelian diseases of high penetrance, is to use a direct filtering strategy in a few families to identify rare or novel variants (Qiao et al., 2016). Many studies identify and validate the candidate variants that were discovered using this approach. They performed WES in a small sample size of family patients to identify potentially related causative genes. The candidate variants were then assessed in a large sample size of case-control population by Sanger sequencing (Mescheriakova et al., 2019; Cetani et al., 2020; Engelbrecht et al., 2020; Froukh et al., 2020).

We undertook WES on eight Uyghur patients from three families with COPD. We showed a DNA-variation map of COPD patients (SNVs, InDels, CNVs, and SVs) at the genetic level, and found 72 SNVs of 55 genes that might be associated with COPD. Scholars have found four genes that might be related to COPD using WES in 49 families with COPD: *DNAH8*, *ALCAM*, *RARS*, and *GBF1*. The main populations they studied were Caucasian and African Americans (Regan et al., 2010; Qiao et al., 2016). Also, the patients they studied were aged <53 years with FEV_1_% ≤40% before bronchodilator inhalation. In our study, we studied a Uyghur population aged >40 years with FEV_1_/FVC <0.70 after bronchodilator inhalation. Those were the differences between ours and previous studies. Furthermore, the final 72 SNVs of 55 genes for COPD identified in our study were different from those determined in previous studies. On the one hand, previous studies selected rare variants, according to the common disease–rare mutation hypothesis (Khera et al., 2018) and most candidate gene methods (Gibson 2012). We did not select rare variants as the research targets. On the other hand, the genetic backgrounds of different ethnicities are different, and even though the same filtering strategy was used, the results might be different (An et al., 2016). Consequently, rs12449210T > A of *HYDIN* and rs777591A > G of *USP34* in our study were found for the first time in a Uyghur COPD population in Kashi.

We evaluated only one HC as a reference; the selected 72 SNVs might contain the SNVs of healthy people. Hence, it was necessary to verify the relationship between these 72 SNVs and COPD in a case-control study.

In the case-control study of two SNVs (rs12449210T > A and rs777591A > G), they were analyzed through genetic models, tobacco smoking, and pulmonary function. Because the PLINK software defaulted a higher mutation frequency to a wild type, in Asia, the “G” mutant frequency of rs777591A > G was higher than “A” (*via* NCBI database: https://www.ncbi.nlm.nih.gov/). Therefore, our data determined “AA” in rs777591A > G (*USP34*) to be a protective factor.

Smoking is the main risk factor for COPD (Vogelmeier et al., 2017); rs777591“AA” showed weak protection. However, in the stratified analysis of smokers and nonsmokers, the protective effect of rs777591“AA” was meaningful only in the nonsmoker group. Based on this analysis, we think rs777591“AA” of USP34 had only a weak protective effect against COPD in the nonsmoker group.

In the present study for the COPD population in Kashi, the allele “A” frequency of rs777591A > G is 35.13%, and the COPD prevalence is 17.01% (Li et al., 2021). In East Asia, the frequency of allele “A” is 32%, but the prevalence of COPD in China (East Asia), Korea (East Asia), and Japan (East Asia) is 13.7% (Wang Y. et al., 2018), 7.82% (Kwon and Kim 2016), and 10.9% (Tan and Ng 2008), respectively. Although South Asia has a higher allele “A” frequency (45%), the prevalence of COPD in countries such as India and Nepal is 4.1% and 18% (Tan and Ng 2008), respectively. Thus, despite the similar frequency of allele “A”, the incidence of COPD varied considerably. This might be because the pathogenesis of COPD was not caused by genes alone, but by the interaction of genetic factors, environmental factors (e.g., tobacco smoking, air pollution, wood and coal consumption) and economic status (Shetty et al., 2021). The protective effect of “A” in rs777591A > G might only be demonstrated in the Kashi COPD population at present, and further validation in different ethnic groups or different regions is required.

*HYDIN* mainly encodes the C2b protein on the central microtubules of motile cilia to adjust the amplitude of the cilia swing and the synergy between cilia (Olbrich et al., 2012). The influence of *HYDIN* on the cilia ultrastructure could lead to abnormal cilia function and, ultimately, reduce the ability to remove foreign bodies (Lechtreck et al., 2008). Some studies show that, in COPD patients, the cilia on the mucosal surface of the respiratory tract are shortened, and cilia motility is reduced, resulting in significant impairment of foreign-body excretion (Hedstrom et al., 2021). Therefore, we speculated that *HYDIN* might have a key role in the occurrence and development of COPD. In this study, the rs12449210T > A of *HYDIN* located in 5′ UTR. *HYDIN* might be related to the abnormal function of respiratory-tract mucosal cilia in COPD, but we showed in a case-control study that *HYDIN* had little effect on the occurrence and development of COPD. This did not mean that *HYDIN* was not associated with COPD in other ethnicities.

Studies show that *USP34* inhibits nuclear factor-kappa B (NF-κB) signal transduction by deubiquitinating and stabilizing the NF-κB inhibitor Iκbα (Li et al., 2020). Studies on the inflammatory cell surface and bronchial epithelial biopsies in COPD cases show that the NF-κB pathway is highly activated (Di Stefano et al., 2002). NF-κB expression has also been found to be increased significantly in the lung tissues of animals with COPD (Yang et al., 2009). Therefore, *USP34* might have a key role in the occurrence and development of COPD. rs777591A > G is located in the intron region at HaploReg v4.1 (ANNOVAR annotated it in 3′UTR of *USP34*). Although rs777591A > G is located in the noncoding regions, genome-wide association studies (GWASs) indicate that only 7% single nucleotide polymorphisms (SNPs) related to complex diseases were located in the coding regions, whereas 93% were in the noncoding regions (Yang et al., 2019). We showed that rs777591“AA” could reduce COPD, so it is a protective factor against COPD. In our *in vitro* study, mRNA and protein expression of USP34 and Iκbα in CSE-treated BEAS-2b cells were obviously higher than those in controls.

We note important limitations of our experimental setting. First, only one non-COPD sample of a COPD family was used to be a control in WES. This may result in containing family-specific variants that are not associated with COPD. It would increase costs and reduce experimental efficiency for case-control validation. Second, the recruited participants were all Uyghurs, and the results might not be representative for ethnic populations. Also, the sample size of the smoking population in the case-control group was small. In addition, although USP34 mRNA and protein expression levels were increased in CSE-treated BEAS-2b cells, the specific role of *USP34* in COPD pathogenesis is unclear.

Due to the above limitations, we will conduct multicenter studies and expand the sample size to further validate our data. Further experiments (including cellular experiments in genetic models, knockout or overexpressed animal experiments) are also required.

## Conclusion

In the present study, WES revealed 72 SNVs, two CNVs, 26 InDels, and 34 SVs in three families with COPD. rs777591 “AA” of *USP34* was a protective factor against COPD, especially in the nonsmoking population. USP34 mRNA and protein expression levels were increased in CSE-treated BEAS-2b cells. Therefore, our data provide new clues for the relationship between USP34 polymorphisms and COPD susceptibility in a Chinese Uyghur population.

## Data Availability

The datasets presented in this study can be found in online repositories. The names of the repository/repositories and accession number(s) can be found below: PRJNA785331

## References

[B1] Abudureheman Z. Li L. Zhong X. Xu J. Gong H. Yilamujiang S. (2021). The rs74794265 SNP of the SREK1 Gene is Associated with COPD in Kashi, China. Copd 16, 2631–2636. 10.2147/COPD.S321150 PMC8453436 34556983

[B2] Abuzhalihan J. Wang Y.-T. Adi D. Ma Y.-T. Fu Z.-Y. Yang Y.-N. (2019). Prevalence of Dyslipidemia in Students from Han, Uygur, and Kazakh Ethnic Groups in a Medical University in Xinjiang, China. Sci. Rep. 9, 19475. 10.1038/s41598-019-55480-5 31857621 PMC6923476

[B3] Adalsteinsson V. A. Ha G. Freeman S. S. Choudhury A. D. Stover D. G. Parsons H. A. (2017). Scalable Whole-Exome Sequencing of Cell-free DNA Reveals High Concordance with Metastatic Tumors. Nat. Commun. 8, 1324. 10.1038/s41467-017-00965-y 29109393 PMC5673918

[B4] An L. Lin Y. Yang T. Hua L. (2016). Exploring the Interaction Among EPHX1, GSTP1, SERPINE2, and TGFB1 Contributing to the Quantitative Traits of Chronic Obstructive Pulmonary Disease in Chinese Han Population. Hum. Genomics. 10, 13. 10.1186/s40246-016-0076-0 27193053 PMC4870730

[B5] Besenbacher S. Sulem P. Helgason A. Helgason H. Kristjansson H. Jonasdottir A. (2016). Multi-nucleotide de novo Mutations in Humans. Plos. Genet. 12, e1006315. 10.1371/journal.pgen.1006315 27846220 PMC5147774

[B6] Cañadas O. Olmeda B. Alonso A. Pérez-Gil J. (2020). Lipid-Protein and Protein-Protein Interactions in the Pulmonary Surfactant System and Their Role in Lung Homeostasis. Int. J. Mol. Sci. 21, 3708. 10.3390/ijms21103708 PMC7279303 32466119

[B7] Cetani F. Pardi E. Aretini P. Saponaro F. Borsari S. Mazoni L. (2020). Whole Exome Sequencing in Familial Isolated Primary Hyperparathyroidism. J. Endocrinol. Invest. 43, 231–245. 10.1007/s40618-019-01107-5 31486992

[B8] de Castro G. S. Simoes E. Lima J. D. C. C. Ortiz-Silva M. Festuccia W. T. Tokeshi F. (2019). Human Cachexia Induces Changes in Mitochondria, Autophagy and Apoptosis in the Skeletal Muscle. Cancers 11, 1264. 10.3390/cancers11091264 PMC6770124 31466311

[B9] Di Stefano A. Caramori G. Oates T. Capelli A. Lusuardi M. Gnemmi I. (2002). Increased Expression of Nuclear Factor- B in Bronchial Biopsies from Smokers and Patients with COPD. Eur. Respir. J. 20, 556–563. 10.1183/09031936.02.00272002 12358328

[B10] Engelbrecht H.-R. Dalvie S. Agenbag G. Stein D. J. Ramesar R. S. (2020). Whole-exome Sequencing in an Afrikaner Family with Bipolar Disorder. J. Affective Disord. 276, 69–75. 10.1016/j.jad.2020.06.045 32697718

[B11] Epi25 Collaborative (2019). Ultra-Rare Genetic Variation in the Epilepsies: A Whole-Exome Sequencing Study of 17,606 Individuals. Am. J. Hum. Genet. 105, 267–282. 10.1016/j.ajhg.2019.05.020 31327507 PMC6698801

[B12] Francioli L. C. Polak P. P. Polak P. P. Koren A. Menelaou A. Chun S. (2015). Genome-wide Patterns and Properties of de novo Mutations in Humans. Nat. Genet. 47, 822–826. 10.1038/ng.3292 25985141 PMC4485564

[B13] Froukh T. Hawwari A. Al Zubi K. (2020). Whole Exome Sequencing Highlights Variants in Association with Keratoconus in Jordanian Families. BMC. Med. Genet. 21, 177. 10.1186/s12881-020-01112-z 32887565 PMC7650294

[B14] Garimella K. V. Iqbal Z. Krause M. A. Campino S. Kekre M. Drury E. (2020). Detection of Simple and Complex de novo Mutations with Multiple Reference Sequences. Genome Res. 30, 1154–1169. 10.1101/gr.255505.119 32817236 PMC7462078

[B15] Gibson G. (2012). Rare and Common Variants: Twenty Arguments. Nat. Rev. Genet. 13, 135–145. 10.1038/nrg3118 22251874 PMC4408201

[B16] Gong H. Ren J. Xu J. Zhong X. Abudureheman Z. Yilamujiang S. (2022). SMAD3 rs36221701 T>C Polymorphism Impacts COPD Susceptibility in the Kashi Population. Gene 808, 145970. 10.1016/j.gene.2021.145970 34547372

[B17] Hedström U. Öberg L. Vaarala O. Dellgren G. Silverborn M. Bjermer L. (2021). Impaired Differentiation of COPD Bronchial Epithelial Cells Grown on Bronchial Scaffolds. Am. J. Respir. Cell. Mol. Biol. 65, 201–213. 10.1165/rcmb.2019-0395OC 33882260 PMC8399573

[B18] Khera A. V. Chaffin M. Aragam K. G. Haas M. E. Roselli C. Choi S. H. (2018). Genome-wide Polygenic Scores for Common Diseases Identify Individuals with Risk Equivalent to Monogenic Mutations. Nat. Genet. 50, 1219–1224. 10.1038/s41588-018-0183-z 30104762 PMC6128408

[B19] Kurkowiak M. Ziętkiewicz E. Witt M. (2015). Recent Advances in Primary Ciliary Dyskinesia Genetics. J. Med. Genet. 52, 1–9. 10.1136/jmedgenet-2014-102755 25351953 PMC4285891

[B20] Kwon H. Kim E. (2016). Factors Contributing to Quality of Life in COPD Patients in South Korea. Copd 11, 103–109. 10.2147/COPD.S90566 PMC4716716 26834467

[B21] Laske K. Shebzukhov Y. V. Grosse-Hovest L. Kuprash D. V. Khlgatian S. V. Koroleva E. P. (2013). Alternative Variants of Human HYDIN Are Novel Cancer-Associated Antigens Recognized by Adaptive Immunity. Cancer Immunol. Res. 1, 190–200. 10.1158/2326-6066.CIR-13-0079 24777681

[B22] Lechtreck K.-F. Delmotte P. Robinson M. L. Sanderson M. J. Witman G. B. (2008). Mutations in Hydin Impair Ciliary Motility in Mice. J. Cell. Biol. 180, 633–643. 10.1083/jcb.200710162 18250199 PMC2234243

[B23] Leung J. M. Tiew P. Y. Mac Aogáin M. Budden K. F. Yong V. F. L. Thomas S. S. (2017). The Role of Acute and Chronic Respiratory Colonization and Infections in the Pathogenesis of COPD. Respirology 22, 634–650. 10.1111/resp.13032 28342288 PMC7169176

[B24] Li L. Zhong X. Zheng A. JianKun C. Budukadeer A. A. Aini P. (2021). Prevalence and Risk Factors of Chronic Obstructive Pulmonary Disease in Kashi Region, Northwestern China. Copd 16, 655–663. 10.2147/COPD.S289620 PMC7981135 33758502

[B25] Li Q. Wang M. Xue H. Liu W. Guo Y. Xu R. (2020). Ubiquitin‐Specific Protease 34 Inhibits Osteoclast Differentiation by RegulatingNF‐κBSignaling. J. Bone. Miner. Res. 35, 1597–1608. 10.1002/jbmr.4015 32212276

[B26] Liu C. Yao X. Li M. Xi Y. Zhao L. (2019). USP39 Regulates the Cell Cycle, Survival, and Growth of Human Leukemia Cells. Biosci. Rep. 39. 10.1042/BSR20190040 PMC6449567 30898977

[B27] Lozano R. Naghavi M. Foreman K. Lim S. Shibuya K. Aboyans V. (2012). Global and Regional Mortality from 235 Causes of Death for 20 Age Groups in 1990 and 2010: a Systematic Analysis for the Global Burden of Disease Study 2010. Lancet 380, 2095–2128. 10.1016/S0140-6736(12)61728-0 23245604 PMC10790329

[B28] Lui T. T. H. Lacroix C. Ahmed S. M. Goldenberg S. J. Leach C. A. Daulat A. M. (2011). The Ubiquitin-specific Protease USP34 Regulates Axin Stability and Wnt/-Catenin Signaling. Mol. Cell Biol. 31, 2053–2065. 10.1128/MCB.01094-10 21383061 PMC3133363

[B29] Meienberg J. Zerjavic K. Keller I. Okoniewski M. Patrignani A. Ludin K. (2015). New Insights into the Performance of Human Whole-Exome Capture Platforms. Nucleic Acids Res. 43, e76. 10.1093/nar/gkv216 25820422 PMC4477645

[B30] Mescheriakova J. Y. Verkerk A. J. Amin N. Uitterlinden A. G. van Duijn C. M. Hintzen R. Q. (2019). Linkage Analysis and Whole Exome Sequencing Identify a Novel Candidate Gene in a Dutch Multiple Sclerosis Family. Mult. Scler. 25, 909–917. 10.1177/1352458518777202 29873607 PMC6545620

[B31] Neofytou E. Tzortzaki E. Chatziantoniou A. Siafakas N. (2012). DNA Damage Due to Oxidative Stress in Chronic Obstructive Pulmonary Disease (COPD). Int. J. Mol. Sci. 13, 16853–16864. 10.3390/ijms131216853 23222732 PMC3546726

[B32] Olbrich H. Schmidts M. Werner C. Onoufriadis A. Loges N. T. Raidt J. (2012). Recessive HYDIN Mutations Cause Primary Ciliary Dyskinesia without Randomization of Left-Right Body Asymmetry. Am. J. Hum. Genet. 91, 672–684. 10.1016/j.ajhg.2012.08.016 23022101 PMC3484652

[B33] Petrovski S. Aggarwal V. Giordano J. L. Stosic M. Wou K. Bier L. (2019). Whole-Exome Sequencing in the Evaluation of Fetal Structural Anomalies: a Prospective Cohort Study. The Lancet 393, 758–767. 10.1016/S0140-6736(18)32042-7 30712878

[B34] Qiao D. Lange C. Beaty T. H. Crapo J. D. Barnes K. C. Bamshad M. (2016). Exome Sequencing Analysis in Severe, Early-Onset Chronic Obstructive Pulmonary Disease. Am. J. Respir. Crit. Care Med. 193, 1353–1363. 10.1164/rccm.201506-1223OC 26736064 PMC4910887

[B35] Regan E. A. Hokanson J. E. Murphy J. R. Make B. Lynch D. A. Beaty T. H. (2010). Genetic Epidemiology of COPD (COPDGene) Study Design. COPD: J. Chronic Obstructive Pulm. Dis. 7, 32–43. 10.3109/15412550903499522 PMC2924193 20214461

[B36] Sauler M. Lamontagne M. Finnemore E. Herazo-Maya J. D. Tedrow J. Zhang X. (2018). The DNA Repair Transcriptome in Severe COPD. Eur. Respir. J. 52, 1701994. 10.1183/13993003.01994-2017 30190272 PMC6422831

[B37] Shapiro A. Davis S. Manion M. Briones K. (2018). Primary Ciliary Dyskinesia (PCD). Am. J. Respir. Crit. Care Med. 198, P3–P4. 10.1164/rccm.1982P3 30004251

[B38] Shetty B. S. P. D'Souza G. andPadukudru Anand M. (2021). Effect of Indoor Air Pollution on Chronic Obstructive Pulmonary Disease (COPD) Deaths in Southern Asia-A Systematic Review and Meta-Analysis. Toxics 9. 10.3390/toxics90400810.3390/toxics9040085 PMC8074040 33923825

[B39] Sy S. M. H. Jiang J. O W. S. Deng Y. Huen M. S. Y. (2013). The Ubiquitin Specific Protease USP34 Promotes Ubiquitin Signaling at DNA Double-Strand Breaks. Nucleic Acids Res. 41, 8572–8580. 10.1093/nar/gkt622 23863847 PMC3794584

[B40] Tan W. C. Ng T. P. (2008). COPD in Asia. Chest 133, 517–527. 10.1378/chest.07-11310.1378/chest.07-1131 18252918 PMC7094310

[B41] Truong L. Zheng Y.-M. Kandhi S. Wang Y.-X. (2021). Overview on Interactive Role of Inflammation, Reactive Oxygen Species, and Calcium Signaling in Asthma, COPD, and Pulmonary Hypertension. Adv. Exp. Med. Biol. 1304, 147–164. 10.1007/978-3-030-68748-9_9 34019268

[B42] Vestbo J. Hurd S. S. Agustí A. G. Jones P. W. Vogelmeier C. Anzueto A. (2013). Global Strategy for the Diagnosis, Management, and Prevention of Chronic Obstructive Pulmonary Disease. Am. J. Respir. Crit. Care Med. 187, 347–365. 10.1164/rccm.201204-0596PP 22878278

[B43] Vogelmeier C. F. Criner G. J. Martinez F. J. Anzueto A. Barnes P. J. Bourbeau J. (2017). Global Strategy for the Diagnosis, Management, and Prevention of Chronic Obstructive Lung Disease 2017 Report. GOLD Executive Summary. Am. J. Respir. Crit. Care Med. 195, 557–582. 10.1164/rccm.201701-0218PP 28128970

[B44] Wang C. C. Xu J. Yang L. Xu Y. Zhang X. Bai C. (2018). Prevalence and Risk Factors of Chronic Obstructive Pulmonary Disease in China (The China Pulmonary Health [CPH] Study): A National Cross-Sectional Study. The Lancet 391, 1706–1717. 10.1016/S0140-6736(18)30841-9 29650248

[B45] Wang J. Sun D. Lu W. Zhang Z. Zhang C. Hu K. (2020). BRIP1 rs10744996C>A Variant Increases the Risk of Chronic Obstructive Pulmonary Disease in the Mongolian Population of Northern China. Exp. Physiol. 105, 1950–1959. 10.1113/EP088210 32851703

[B46] Wang X. Huang L. Jiang S. Cheng K. Wang D. Luo Q. (2021). Testosterone Attenuates Pulmonary Epithelial Inflammation in Male Rats of COPD Model through Preventing NRF1-Derived NF-κB Signaling. J. Mol. Cell. Biol. 13, 128–140. 10.1093/jmcb/mjaa079 33475136 PMC8104951

[B47] Wang Y. Zhou Q. Dong L. Xiong M. Jiang H. Guo M. (2018). The Effects of CXCL10 Polymorphisms on COPD Susceptibility. Mol. Genet. Genomics. 293, 649–655. 10.1007/s00438-017-1408-z 29285564

[B48] Xu S. Jin L. (2008). A Genome-wide Analysis of Admixture in Uyghurs and a High-Density Admixture Map for Disease-Gene Discovery. Am. J. Hum. Genet. 83, 322–336. 10.1016/j.ajhg.2008.08.001 18760393 PMC2556439

[B49] Yang S.-R. Yao H. Rajendrasozhan S. Chung S. Edirisinghe I. Valvo S. (2009). RelB Is Differentially Regulated by IκB Kinase-α in B Cells and Mouse Lung by Cigarette Smoke. Am. J. Respir. Cell. Mol. Biol. 40, 147–158. 10.1165/rcmb.2008-0207OC 18688039 PMC2633139

[B50] Yang T. Li J. Wen Y. Tan T. Yang J. Pan J. (2019). LINC00673 rs11655237 C>T Polymorphism Impacts Hepatoblastoma Susceptibility in Chinese Children. Front. Genet. 10, 506. 10.3389/fgene.2019.00506 31178901 PMC6544040

[B51] Zhang W. Li Y. Lin J. Abduryim A. Zhao J. (2018). Cariogenicity of Candida Albicans of Distinct Genotypes Among 3-5-Year-Old Uygur Children in Kashgar, China- a Case-Control Study. BMC. Oral Health 18, 203. 10.1186/s12903-018-0658-4 30518349 PMC6282366

[B52] Zhang Z. Wang J. Chen L. Chen X. Sun G. Zhong N. (2014). Impact of Haze and Air Pollution-Related Hazards on Hospital Admissions in Guangzhou, China. Environ. Sci. Pollut. Res. 21, 4236–4244. 10.1007/s11356-013-2374-6 24306726

[B53] Zhang Z. Wang J. Zheng Z. Chen X. Xu G. Chen S. (2020). A Protective Polymorphism in MMP16 , Improved Blood Gas Levels, and Chronic Obstructive Pulmonary Diseases: Family and Two Population‐based Studies. Hum. Mutat. 41, 1280–1297. 10.1002/humu.24013 32196811

