# Supplementary material for: Whole-Exome Sequencing Implicates the USP34 rs777591A > G Intron Variant in Chronic Obstructive Pulmonary Disease in a Kashi Cohort
Source: Front Cell Dev Biol. 2022 Feb 7;9:792027. doi: 10.3389/fcell.2021.792027 (PMC8859106; doi:10.3389/fcell.2021.792027)
Supplement: Supplementary file 1 [file DataSheet1.doc]

Supplementary Material

1. **Supplementary Methods**

**1. 1. WES**

After realizing DNA fragmentation to the target-length range, a bioanalyzer (2100 series; Agilent Technologies, Santa, Clara, CA, USA) was used for quality control. Then, the SureSelect Human All Exon V6 kit (Agilent Technologies) was used to build a DNA library.

After polymerase chain reaction (PCR) amplification, we quantified the constructed DNA library by Qubit™ 2.0 DNA Broad Range Assay (Invitrogen, Carlsbad, CA, USA). Finally, the bioanalyzer (2100 series; Agilent) was used to detect the fragment size of the DNA library. Qualified samples were sequenced by the HiSeq™ X-Ten system (Illumina, San Diego, CA, USA).

**1. 2. Variants selection**

The original reads (paired ends) were evaluated and filtered by sequencing. Valid sequencing reads were matched to the reference genome (GRCH38/hg38). Then, ANNOVAR (<https://annovar.openbioinformatics.org/>) (Wang et al., 2010) and Circos (<http://mkweb.bcgsc.ca/circos>) (Krzywinski et al., 2009) were used for variants annotation and overview of global variation. Simultaneously, SNVs were selected using the dbNSFP database (<http://database.liulab.science/dbNSFP>) (Liu et al., 2020) to evaluate the conservation and pathogenicity of amino acids by calculating the corresponding scores, SIFT (https://sift.bii.a-star.edu.sg/) and PolyPhen-2 (<http://genetics.bwh.harvard.edu/pph2>). The SIFT scores less than 0.05 were harmful variants, and PolyPhen-2 scores more than 0.453 were harmful variants. CNVs were selected by using Control-FREEC software (Boeva et al., 2012). CNVs within 10k was marked according to the gap region and segmental duplication region of the genome. Annotation of CNVs was used SnpEff (Cingolani et al., 2012). BreakDancer (Chen et al., 2009) was used to undertake SV detection on samples. Then, we marked SVs within 10k according to the gap region and segmental duplication region of the genome. ANNOVAR was used for SVs annotation.

**1.3. Filtering strategy for SNVs, InDels, CNVs, and SVs**

The filtering strategy for SNVs, InDels, CNVs, and SVs was as followed. First, we recorded the common variants of each family as “family B_same”, “family C_same”, and “family N_same”. Then, we recorded different variants between “N2” (HC) and “family N_same” as “family N_diff”. Then, we took the intersection of “family B_same”, “family C_same”, and “family N_diff” to obtain the different variants we wished to analyze. We wished to compare the variants between all samples and to screen-out the variants located in the functional gene region. Hence, we reserved the variants in the: untranslated region (UTR; upstream and downstream); variable splicing region; non-synonymous mutations and frameshift mutations of the exon region; the change of stop codons and unknown types; the exon region of non-coding RNA. Additionally, we also reserved SNVs with different annotation results form those in the database.

**1. 4. Sanger sequencing in the case–control study**

SNVs were genotyped using SNPscan™ (Center for Genetic & Genomic Analysis, Genesky Biotechnologies, Shanghai, China) for sequencing and analyses with a DNA analyzer (ABI3730XL; Applied Biosystems, Foster City, CA, USA) and GeneMapper™ 4.1 (Applied Biosystems).

**1. 5. Enrichment analyses using the Gene Ontology (GO) and Kyoto Encyclopedia of Genes and Genomes (KEGG) databases**

Enrichment analyses of all common mutant genes in the three families was done using the GO database ([www.geneontology.org/](http://www.geneontology.org/)) (The Gene Ontology 2019). Analyses of pathway enrichment of genes was undertaken using the KEGG database ([www.genome.jp/kegg/](http://www.genome.jp/kegg/)) (Kanehisa et al., 2017). We used KEGG DISEASE (www.genome.jp/kegg/disease/) (Kanehisa et al., 2010), OMIM ([omim.org](https://omim.org/)) (Amberger and Hamosh 2017), GAD (geneticassociationdb.nih.gov) (Becker et al., 2004), and NHGRI GWAS Catalog ([www.genome.gov/gwastudies](http://www.genome.gov/gwastudies)) (Hindorff et al., 2009) to detect whether the mutation was in the relevant database.

**1. 6. Chemicals and reagents**

Antibodies against USP34 (catalog number: ab91617) and nuclear factor of kappa light polypeptide gene enhancer in B-cells inhibitor, alpha (Iκbα; ab12134) were purchased from Abcam (Cambridge, UK). Polyvinylidene difluoride membranes were bought from Bio-Rad Laboratories (Hercules, CA, USA). TRIzol™ Reagent was from Invitrogen. Goat anti-mouse IgG (H+L) and goat anti-rabbit IgG (H+L) were purchased from Beijing Odyssey Chemicals (Beijing, China). Fetal bovine serum (FBS) and Dulbecco's modified Eagle’s medium (DMEM) were obtained from Gibco (Grand Island, NY, USA).

**1. 7. Preparation of cigarette smoke extract (CSE)**

CSE was prepared using filter cigarettes (Furongwang; Changde Tobacco Company, Hunan, China). CSE was prepared freshly with three filtered cigarettes into 30 mL DMEM media within 30 min prior to treatments. The pH of the suspension was changed to pH 7.4, and passed through a 0.22-µm filter to remove bacteria. CSE preparations were split into portions and preserved at 4°C, which were deemed to be 100%, and diluted based on the experimental scheme.

**1. 8. Cell culture and CSE treatment**

The bronchial epithelial beas-2b (BEAS-2b) cell line was purchased from the Cell Bank of the Chinese Academy of Sciences (Shanghai, China). DMEM containing 10% FBS, 100 µg/mL streptomycin, and 100 U/mL penicillin was used to culture cells in a humidified incubator at 37℃ in an atmosphere of 5% CO_2_. For CSE exposure, BEAS-2b cells were cultured in six-well plates until the cell density reached 60%–70%. Then, 0%–3% CSE was used to treat cells for 24 h in 2 mL of medium with 10% FBS. We added fresh CSE suspensions uniformly to the cell-culture media. After CSE exposure, cells were washed twice with phosphate-buffered saline, and we collected the cell lysates for western blotting.

**1. 9. Western blotting**

Lysis buffer (25 mM Tris-HCl, pH 7.6, 150 mM NaCl, 1% NP-40, 1% sodium deoxycholate, and 0.1% sodium dodecyl sulfate) was used to lyse cells for 30 min at 4℃. Then, the cell lysates were centrifuged at 14,000 × g for 15 min at 4℃. A bicinchoninic acid kit (Thermo Scientific, Waltham, MA, USA) were used to measure the protein concentration. We used sodium dodecyl sulfate–polyacrylamide gel electrophoresis to resolve proteins and western blotting to measure protein expression.

**1. 10. RNA isolation and real-time RT-qPCR**

Cells were washed by cold phosphate-buffered saline and lysed in TRIzol Reagent. RNA was changed into complimentary-DNA. Expression of USP34 and Iκbα was analyzed by LightCycler® 480 SYBR Green I Master (Roche, Basel, Switzerland). Primer pairs (forward and reverse, respectively) were 5′-ATCCTCACCCTGAAGTACCCC-3′ and 5′-CCACACGCAGCTCATTGTAGAAGG-3′ for β-actin; 5′-CATCATCCATGAAGAAAAGGCACT-3′ and 5′-TCTCGGAGCTCAGGATCACA-3′ for Iκbα; 5′-AACTCAAGAAAGTAACATATTAGGGG-3′ and 5′-CTTCATGAGAGAAAGGCCAT-3′ for USP34. The 2^−Ct△△^ method was used to measure gene expression in reference to that of β-actin.

**References**

Amberger, J. S.,Hamosh, A. (2017). Searching Online Mendelian Inheritance in Man (OMIM): A Knowledgebase of Human Genes and Genetic Phenotypes. *Curr Protoc Bioinformatics*. 58, 1 2 1-1 2 12. doi: 10.1002/cpbi.27

Becker, K. G., Barnes, K. C., Bright, T. J., andWang, S. A. (2004). The genetic association database. *Nat Genet*. 36, 431-432. doi: 10.1038/ng0504-431

Boeva, V., Popova, T., Bleakley, K., Chiche, P., Cappo, J., Schleiermacher, G., et al. (2012). Control-FREEC: a tool for assessing copy number and allelic content using next-generation sequencing data. *Bioinformatics*. 28, 423-425. doi: 10.1093/bioinformatics/btr670

Chen, K., Wallis, J. W., McLellan, M. D., Larson, D. E., Kalicki, J. M., Pohl, C. S., et al. (2009). BreakDancer: an algorithm for high-resolution mapping of genomic structural variation. *Nat Methods*. 6, 677-681. doi: 10.1038/nmeth.1363

Cingolani, P., Platts, A., Wang le, L., Coon, M., Nguyen, T., Wang, L., et al. (2012). A program for annotating and predicting the effects of single nucleotide polymorphisms, SnpEff: SNPs in the genome of Drosophila melanogaster strain w1118; iso-2; iso-3. *Fly (Austin)*. 6, 80-92. doi: 10.4161/fly.19695

Hindorff, L. A., Sethupathy, P., Junkins, H. A., Ramos, E. M., Mehta, J. P., Collins, F. S., et al. (2009). Potential etiologic and functional implications of genome-wide association loci for human diseases and traits. *Proc Natl Acad Sci U S A*. 106, 9362-9367. doi: 10.1073/pnas.0903103106

Kanehisa, M., Furumichi, M., Tanabe, M., Sato, Y., andMorishima, K. (2017). KEGG: new perspectives on genomes, pathways, diseases and drugs. *Nucleic Acids Res*. 45, D353-D361. doi: 10.1093/nar/gkw1092

Kanehisa, M., Goto, S., Furumichi, M., Tanabe, M., andHirakawa, M. (2010). KEGG for representation and analysis of molecular networks involving diseases and drugs. *Nucleic Acids Res*. 38, D355-360. doi: 10.1093/nar/gkp896

Krzywinski, M., Schein, J., Birol, I., Connors, J., Gascoyne, R., Horsman, D., et al. (2009). Circos: an information aesthetic for comparative genomics. *Genome Res*. 19, 1639-1645. doi: 10.1101/gr.092759.109

Liu, X., Li, C., Mou, C., Dong, Y., andTu, Y. (2020). dbNSFP v4: a comprehensive database of transcript-specific functional predictions and annotations for human nonsynonymous and splice-site SNVs. *Genome Med*. 12, 103. doi: 10.1186/s13073-020-00803-9

The Gene Ontology, C. (2019). The Gene Ontology Resource: 20 years and still GOing strong. *Nucleic Acids Res*. 47, D330-D338. doi: 10.1093/nar/gky1055

Wang, K., Li, M., andHakonarson, H. (2010). ANNOVAR: functional annotation of genetic variants from high-throughput sequencing data. *Nucleic Acids Res*. 38, e164. doi: 10.1093/nar/gkq603

1. **Supplementary Tables**

**Table S1. Subjects basic information in three families**

| **Families^a^** | **ID^a^** | **Sex** | **Area in live** | **Human species** | **Diagnose** |
| --- | --- | --- | --- | --- | --- |
| **B** | B1 | Female | Kashi,Xingjiang,China | Chinese Uyghur | COPD |
|  | B2 | Male | Kashi,Xingjiang,China | Chinese Uyghur | COPD |
|  | B3 | Male | Kashi,Xingjiang,China | Chinese Uyghur | COPD |
| **C** | C1 | Female | Kashi,Xingjiang,China | Chinese Uyghur | COPD |
|  | C2 | Female | Kashi,Xingjiang,China | Chinese Uyghur | COPD |
| **N** | N1 | Male | Kashi,Xingjiang,China | Chinese Uyghur | COPD |
|  | N2 | Female | Kashi,Xingjiang,China | Chinese Uyghur | None COPD |
|  | N3 | Female | Kashi,Xingjiang,China | Chinese Uyghur | COPD |
|  | N4 | Female | Kashi,Xingjiang,China | Chinese Uyghur | COPD |

a: Families and ID were from Figure 1.

**Table S2. The 72 SNVs after filtering of COPD family subjects**

| **No** | **rs** | **Gene** | **Chr** | **Position** | **Ref** | **Alt** | **Alt allele frequency** | |  | **MAF** | |
| --- | --- | --- | --- | --- | --- | --- | --- | --- | --- | --- | --- |
|  |  |  |  |  |  |  | **general population^a^** | **region population^b^** |  | **Case** | **Control** |
| 1 | rs4880338 | *JAKMIP3* | 10 | 132168178 | G | A | 0.94 | 0.75 |  | 0.27 | 0.26 |
| 2 | rs7414943 | *DRAXIN* | 1 | 11722757 | G | A | 0.40 | 0.43 |  | 0.43 | 0.42 |
| 3 | rs3741212 | *IGF2-AS* | 11 | 2140628 | A | G | 0.92 | 0.80 |  | 0.20 | 0.20 |
| 4 | rs15783 | *ANO3* | 11 | 26565254 | G | A | 0.42 | 0.60 |  | 0.38 | 0.41 |
| 5 | rs6587553 | *BNIPL* | 1 | 151046704 | A | G | 0.92 | 0.89 |  | 0.12 | 0.13 |
| 6 | rs4971007 | *SEMA6C* | 1 | 151135661 | T | G | 0.92 | 0.88 |  | 0.11 | 0.13 |
| 7 | rs4233367 | *ADAMTS4* | 1 | 161193247 | T | C | 0.91 | 0.73 |  | 0.27 | 0.26 |
| 8 | rs7112561 | *GVINP1* | 11 | 6717878 | G | A | 0.52 | 0.54 |  | 0.45 | 0.46 |
| 9 | rs12284429 | *GVINP1* | 11 | 6718176 | A | G | 0.53 | 0.54 |  | 0.45 | 0.46 |
| 10 | rs1044782 | *PGM2L1* | 11 | 74336638 | A | G | 0.78 | 0.69 |  | 0.30 | 0.32 |
| 11 | rs2503701 | *FAAP20* | 1 | 2189679 | C | T | 0.87 | 0.79 |  | 0.22 | 0.20 |
| 12 | rs646356 | *MROH7* | 1 | 54702100 | T | A | 0.95 | 0.87 |  | 0.13 | 0.13 |
| 13 | rs4072393 | *TMC7* | 16 | 19030273 | G | A | 0.78 | 0.68 |  | 0.32 | 0.33 |
| 14 | rs12449210 | *HYDIN* | 16 | 71230620 | T | A | 0.35 | 0.45 |  | 0.47 | 0.44 |
| 15 | rs2868591 | *NPIPB15* | 16 | 74391650 | A | G | 0.73 | NA |  | NA | NA |
| 16 | rs6500493 | *PIEZO1* | 16 | 88737574 | C | G | 0.90 | 0.78 |  | 0.22 | 0.22 |
| 17 | rs4785755 | *CENPBD1* | 16 | 89971420 | G | A | 0.78 | 0.77 |  | 0.22 | 0.24 |
| 18 | rs346822 | *SLC52A1* | 17 | 5034280 | T | C | 0.83 | 0.90 |  | 0.11 | 0.09 |
| 19 | rs1061728 | *ADGRL4* | 1 | 78889911 | A | G | 0.65 | 0.73 |  | 0.28 | 0.26 |
| 20 | rs5516 | *KLK1* | 19 | 50820217 | C | G | 0.74 | 0.71 |  | 0.28 | 0.30 |
| 21 | rs161931 | *ZNF578* | 19 | 52511093 | G | A | 0.66 | NA |  | NA | NA |
| 22 | rs8107444 | *ZNF28* | 19 | 52800274 | A | G | 0.89 | NA |  | NA | NA |
| 23 | rs1132274 | *RRBP1* | 20 | 17615510 | C | A | 0.51 | 0.30 |  | 0.30 | 0.33 |
| 24 | rs11960 | *RRBP1* | 20 | 17619712 | G | A | 0.28 | 0.57 |  | 0.44 | 0.42 |
| 25 | rs214832 | *TGM3* | 20 | 2341019 | T | C | 0.94 | 0.76 |  | 0.24 | 0.23 |
| 26 | rs8788 | *GART* | 21 | 33524806 | C | T | 0.99 | 0.84 |  | 0.16 | 0.17 |
| 27 | rs922984 | *TTN* | 2 | 178751160 | T | C | 0.83 | 0.84 |  | 0.18 | 0.14 |
| 28 | rs7585334 | *TTN* | 2 | 178756224 | C | T | 0.76 | 0.84 |  | 0.19 | 0.14 |
| 29 | rs2291310 | *TTN* | 2 | 178759031 | C | T | 0.77 | 0.84 |  | 0.18 | 0.14 |
| 30 | rs2291311 | *TTN* | 2 | 178764734 | C | T | 0.77 | 0.84 |  | 0.18 | 0.14 |
| 31 | rs3748993 | *NYAP2* | 2 | 225627054 | A | C | 0.82 | 0.86 |  | 0.14 | 0.13 |
| 32 | rs2289235 | *ITM2C* | 2 | 230873453 | G | A | 0.87 | 0.66 |  | 0.33 | 0.35 |
| 33 | rs2277841 | *FOXRED2* | 22 | 36504224 | T | C | 0.26 | 0.47 |  | 0.47 | 0.48 |
| 34 | rs1243166 | *SERPINA1* | 14 | 94377481 | A | G | 0.64 | 0.64 |  | 0.35 | 0.37 |
| 35 | rs1051052 | *SERPINA1* | 14 | 94377595 | A | G | 0.67 | 0.56 |  | 0.43 | 0.44 |
| 36 | rs12628964 | *MOV10L1* | 22 | 50090140 | A | T | 0.32 | 0.30 |  | 0.29 | 0.30 |
| 37 | rs9617066 | *MOV10L1* | 22 | 50092072 | A | T | 0.23 | 0.30 |  | 0.30 | 0.31 |
| 38 | rs3810971 | *MOV10L1* | 22 | 50108237 | C | T | 0.32 | 0.30 |  | 0.29 | 0.30 |
| 39 | rs2340601 | *MOV10L1* | 22 | 50134044 | G | A | 0.33 | 0.30 |  | 0.29 | 0.31 |
| 40 | rs2272837 | *MOV10L1* | 22 | 50144197 | A | G | 0.61 | 0.40 |  | 0.38 | 0.42 |
| 41 | rs777591 | *USP34* | 2 | 61190482 | A | G | 0.68 | 0.65 |  | 0.33 | 0.38 |
| 42 | rs1054565 | *TAF1B* | 2 | 9910831 | A | G | 0.50 | 0.53 |  | 0.48 | 0.46 |
| 43 | rs1820965 | *TAF1B* | 2 | 9919641 | G | T | 0.53 | 0.54 |  | 0.47 | 0.44 |
| 44 | rs1801726 | *CASR* | 3 | 122284985 | G | C | 0.98 | 0.97 |  | 0.03 | 0.04 |
| 45 | rs777241 | *TXNRD3* | 3 | 126611067 | A | G | 0.86 | 0.94 |  | 0.07 | 0.06 |
| 46 | rs182398 | *FGD5* | 3 | 14921441 | T | C | 0.89 | 0.91 |  | 0.10 | 0.08 |
| 47 | rs1685637 | *FGD5* | 3 | 14923314 | C | T | 0.95 | 0.91 |  | 0.10 | 0.08 |
| 48 | rs661301 | *ZFYVE28* | 4 | 2304326 | A | G | 0.73 | 0.84 |  | 0.16 | 0.16 |
| 49 | rs35652124 | *NFE2L2* | 2 | 177265345 | T | C | 0.59 | 0.48 |  | 0.48 | 0.48 |
| 50 | rs1108512 | *RBM24* | 6 | 17282184 | G | C | 0.74 | 0.74 |  | 0.27 | 0.26 |
| 51 | rs926529 | *ACOT13* | 6 | 24687537 | A | G | 0.81 | 0.61 |  | 0.25 | 0.24 |
| 52 | rs6938590 | *SERPINB6* | 6 | 2971027 | T | C | 0.98 | NA |  | NA | NA |
| 53 | rs6721961 | *NFE2L2* | 2 | 177265309 | T | G | 0.78 | 0.78 |  | 0.22 | 0.21 |
| 54 | rs491152 | *MMP1* | 11 | 102795312 | A | G | 0.85 | 0.89 |  | 0.10 | 0.12 |
| 55 | rs1049674 | *ASNS* | 7 | 97859257 | A | T | 0.91 | 0.80 |  | 0.21 | 0.18 |
| 56 | rs2721166 | *CYHR1* | 8 | 144464925 | C | T | 0.74 | 0.95 |  | 0.06 | 0.05 |
| 57 | rs2620644 | *CYHR1* | 8 | 144464965 | G | A | 0.78 | 0.95 |  | 0.06 | 0.05 |
| 58 | rs2620645 | *CYHR1* | 8 | 144465013 | A | C | 0.77 | 0.95 |  | 0.06 | 0.05 |
| 59 | rs2721190 | *RECQL4* | 8 | 144517130 | A | G | 0.76 | 0.95 |  | 0.05 | 0.04 |
| 60 | rs62579871 | *ESP33* | 9 | 136250864 | G | A | 0.63 | 0.87 |  | 0.13 | 0.14 |
| 61 | rs35870000 | *FREM1* | 9 | 14801712 | C | A | 0.19 | 0.29 |  | 0.29 | 0.29 |
| 62 | rs3738368 | *SLFNL1* | 1 | 41020230 | C | G | 0.14 | 0.25 |  | 0.25 | 0.25 |
| 63 | rs1800517 | *COL4A4* | 2 | 227051116 | G | A | 0.44 | 0.51 |  | 0.47 | 0.50 |
| 64 | rs3740423 | *MKI67* | 10 | 128107632 | T | A | 0.02 | 0.22 |  | 0.21 | 0.23 |
| 65 | rs5006885 | *OR51B6* | 11 | 5352012 | T | G | 0.06 | 0.15 |  | 0.15 | 0.14 |
| 66 | rs5006884 | *OR51B6* | 11 | 5352021 | C | T | 0.06 | 0.15 |  | 0.15 | 0.14 |
| 67 | rs5024042 | *OR51B6* | 11 | 5352332 | C | A | 0.06 | 0.14 |  | 0.15 | 0.14 |
| 68 | rs7252027 | *EID2* | 19 | 39540064 | C | T | 0.25 | 0.26 |  | 0.28 | 0.25 |
| 69 | rs1799782 | *XRCC1* | 19 | 43553422 | G | A | 0.32 | 0.15 |  | 0.16 | 0.14 |
| 70 | rs1048621 | *SDCBP2* | 20 | 1312402 | G | A | 0.01 | 0.11 |  | 0.11 | 0.10 |
| 71 | rs6135876 | *OTOR* | 20 | 16748493 | T | C | 0.30 | 0.26 |  | 0.25 | 0.27 |
| 72 | rs4339026 | *BPIFB4* | 20 | 33083793 | A | G | 0.13 | 0.16 |  | 0.23 | 0.18 |

Ref: Reference allele; Alt: alter allele (mutant allele).

a: The allele frequency of general population was from East Asian population in dbGaP database.

b: The alter allele frequency of region population = mutant allele/total allele.

NA: Not Applicable. Genotyping failed.

**Table S3. The 26 InDels after filtering of COPD family subjects**

| **Chr** | **Start** | **End** | **REF** | **ALT** | **Region** | **Gene** |
| --- | --- | --- | --- | --- | --- | --- |
| chr1 | 35565754 | 35565754 | T | - | UTR3 | *NCDN* |
| chr1 | 42830498 | 42830499 | CT | - | exonic | *ERMAP* |
| chr1 | 84574316 | 84574324 | GCAGCGCCA | - | exonic | *CTBS* |
| chr2 | 178614391 | 178614391 | - | T | ncRNA_exonic | *TTN-AS1* |
| chr3 | 11807450 | 11807453 | CACT | - | exonic | *TAMM41* |
| chr3 | 113249862 | 113249864 | CCT | - | exonic | *BOC* |
| chr3 | 129418380 | 129418380 | A | - | exonic | *EFCAB12* |
| chr4 | 30339959 | 30339960 | GT | - | intergenic | *LOC105374562,PCDH7* |
| chr6 | 42929620 | 42929622 | TGC | - | exonic | *CNPY3,CNPY3-GNMT* |
| chr6 | 136525979 | 136525979 | T | - | UTR5 | *MAP7* |
| chr7 | 72833891 | 72833891 | T | - | ncRNA_exonic | *SBDSP1* |
| chr10 | 77008012 | 77008023 | TGTAGACTGCAT | - | UTR3 | *KCNMA1* |
| chr10 | 91630215 | 91630215 | - | A | exonic | *PPP1R3C* |
| chr12 | 7080481 | 7080481 | T | - | UTR3 | *C1R* |
| chr12 | 40406131 | 40406131 | - | GTCAGGATG | exonic | *MUC19* |
| chr12 | 40416307 | 40416307 | - | T | ncRNA_exonic | *LOC105369736* |
| chr12 | 40516467 | 40516467 | G | - | exonic | *MUC19* |
| chr12 | 50353588 | 50353588 | C | - | exonic | *FAM186A* |
| chr13 | 44574730 | 44574732 | CCA | - | exonic | *TSC22D1* |
| chr13 | 48037782 | 48037782 | - | GGAGTC | exonic | *NUDT15* |
| chr16 | 31602305 | 31602305 | T | - | intergenic | *YBX3P1,CLUHP3* |
| chr16 | 57813723 | 57813724 | TG | - | exonic | *LOC388282* |
| chr16 | 57813726 | 57813731 | AGGTGG | - | exonic | *LOC388282* |
| chr18 | 649944 | 649945 | TT | - | UTR3 | *CLUL1,TYMSOS* |
| chr22 | 50090067 | 50090067 | - | GGTG | UTR5 | *MOV10L1* |
| chr22 | 50090068 | 50090068 | - | CGGCG | UTR5 | *MOV10L1* |

**Table S4. The two CNVs after filtering of COPD family subjects**

| **Chr** | **Start** | **End** | **Copy number** | **Status** |
| --- | --- | --- | --- | --- |
| chr1 | 146989595 | 146985707 | 0 | loss |
| chr6 | 105929 | 146631 | 0 | loss |

**Table S5. The 34 SVs after filtering of COPD family subjects**

| **Chr1** | **Pos1** | **Chr2** | **Pos2** | **Type** | **numreads** | **Region1** | **Gene1** | **Region2** | **Gene2** |
| --- | --- | --- | --- | --- | --- | --- | --- | --- | --- |
| chr1 | 43621721 | chr1 | 43622064 | ITX | 85 | intronic | *PTPRF* | UTR3 | *PTPRF* |
| chr1 | 46277612 | chr1 | 46278187 | ITX | 86 | intronic | *RAD54L* | exonic | *RAD54L* |
| chr1 | 228022468 | chr1 | 228022898 | ITX | 72 | intronic | *WNT3A* | exonic | *WNT3A* |
| chr1 | 204155404 | chr1 | 204156275 | ITX | 97 | intronic | *REN* | exonic | *REN* |
| chr1 | 179085330 | chr1 | 179085874 | ITX | 92 | intronic | *TOR3A* | exonic | *TOR3A* |
| chr10 | 80274330 | chr10 | 80274631 | ITX | 90 | intronic | *MAT1A* | exonic | *MAT1A* |
| chr10 | 100993105 | chr10 | 100993536 | ITX | 83 | intronic | *C10orf2* | UTR3 | *C10orf2* |
| chr11 | 7651507 | chr11 | 7651827 | ITX | 65 | intronic | *PPFIBP2* | exonic | *PPFIBP2* |
| chr11 | 56462262 | chr11 | 56463318 | ITX | 100 | downstream | *OR5M9* | exonic | *OR5M9* |
| chr12 | 112947681 | chr12 | 112948054 | ITX | 87 | intronic | *OAS3* | exonic | *OAS3* |
| chr12 | 112151545 | chr12 | 112152090 | ITX | 101 | intronic | *TRAFD1* | exonic | *TRAFD1* |
| chr12 | 895972 | chr12 | 896674 | ITX | 85 | intronic | *WNK1* | exonic | *WNK1* |
| chr14 | 23969819 | chr14 | 23970240 | ITX | 97 | Upstream,  downstream | *DHRS4L2,*  *DHRS4* | UTR5 | *DHRS4L2* |
| chr15 | 41865377 | chr15 | 41866614 | ITX | 114 | UTR3 | *SPTBN5* | intronic | *SPTBN5* |
| chr15 | 39587135 | chr15 | 39587518 | ITX | 73 | intronic | *THBS1* | exonic | *THBS1* |
| chr17 | 40188557 | chr17 | 40188950 | ITX | 82 | intronic | *RAPGEFL1* | exonic | *RAPGEFL1* |
| chr17 | 42773114 | chr17 | 42773907 | ITX | 89 | upstream | *VPS25* | intronic | *VPS25* |
| chr19 | 5130900 | chr19 | 5131431 | ITX | 93 | intronic | *KDM4B* | exonic | *KDM4B* |
| chr19 | 40629164 | chr19 | 40629640 | ITX | 110 | intronic | *LTBP4* | UTR3 | *LTBP4* |
| chr19 | 10315017 | chr19 | 10316228 | ITX | 98 | intronic | *FDX1L* | UTR3 | *RAVER1* |
| chr19 | 49614303 | chr19 | 49614957 | ITX | 69 | intronic | *PRR12* | exonic | *PRR12* |
| chr19 | 45302124 | chr19 | 45302665 | ITX | 101 | intronic | *MARK4* | exonic | *MARK4* |
| chr2 | 108767869 | chr2 | 108768312 | ITX | 125 | exonic | *RANBP2* | exonic | *RANBP2* |
| chr2 | 166432213 | chr2 | 166432750 | ITX | 73 | intronic | *SCN7A* | exonic | *SCN7A* |
| chr20 | 63975359 | chr20 | 63975837 | ITX | 69 | UTR3 | *SAMD10* | intronic | *SAMD10* |
| chr20 | 45899360 | chr20 | 45899667 | ITX | 83 | intronic | *PLTP* | exonic | *PLTP* |
| chr3 | 50378597 | chr3 | 50379186 | ITX | 79 | intronic | *CACNA2D2* | exonic | *CACNA2D2* |
| chr4 | 140497943 | chr4 | 140498341 | ITX | 88 | intronic | *MGAT4D* | UTR5 | *MGAT4D* |
| chr4 | 55124955 | chr4 | 55125271 | ITX | 80 | intronic | *KDR* | exonic | *KDR* |
| chr6 | 159748248 | chr6 | 159748598 | ITX | 72 | exonic | *WTAP* | exonic | *WTAP* |
| chr7 | 155811392 | chr7 | 155812177 | ITX | 95 | UTR5 | *SHH* | UTR5 | *SHH* |
| chr9 | 133205557 | chr9 | 133206398 | ITX | 103 | UTR3 | *OBP2B* | exonic | *OBP2B* |
| chr9 | 136381574 | chr9 | 136382057 | ITX | 89 | intronic | *SNAPC4* | exonic | *SNAPC4* |
| chr9 | 122628516 | chr9 | 122629556 | ITX | 104 | UTR3 | *OR1B1* | UTR5 | *OR1B1* |

**Table S6. Smoking exposure history for smokers (former and current) in case and control groups**

| **Variables** | **Case**  **n = 124 (%)** | | **Control**  **n = 69 (%)** | **P** |
| --- | --- | --- | --- | --- |
| **Cigarettes per day (former^a^ and current^b^ smokers), n (%)** |  | |  | 0.258 |
| Light smokers (< 20) | 65 (52.42) | | 42 (60.87) |  |
| Heavy smokers (≥20) | 59 (47.58) | | 27 (39.13) |  |
| **Cumulative quantity of active smoking (pack years), n (%)** |  | |  | 0.748 |
| ≤15 | 42 (33.87) | | 21 (30.43) |  |
| 15-30 | 47 (37.90) | | 30 (43.48) |  |
| > 30 | 35 (28.23) | | 18 (26.09) |  |
| **Quitting smoking years (former smokers), n (%)** |  | |  | 0.336 |
| ≤5 | 14 (60.87) | | 10 (58.82) |  |
| 5-10 | 7 (30.43) | | 6 (35.29) |  |
| > 10 | 2 (8.70) | 1 (5.89) | |  |

a: The number of former smokers in case group: 23; in control group: 17.

b: The number of current smokers in case group: 101; in control group: 52.

**Table S7. Analysis of genotypes for rs12449210 of *HYDIN* among non-smokers and smokers**

| **SNV** | **Model** | **Genotype** | **Non-smokers** | | | |  | **Smokers** | | | |  | **Heterogeneity test** | |
| --- | --- | --- | --- | --- | --- | --- | --- | --- | --- | --- | --- | --- | --- | --- |
|  |  |  | **Case** | **Control** | **OR (95%CI)^a^** | **P^a^** |  | **Case** | **Control** | **OR (95%CI)^a^** | **P^a^** |  | ***χ*2** | **P** |
| **rs12449210**  **of *HYDIN*** | Genotype | A/A | 79 (21.35%) | 97 (21.00%) | 1.163 (0.7703–1.755) | 0.4731 |  | 29 (23.58%) | 16 (23.53%) | 1.037 (0.4449–2.416) | 0.9334 |  | 0.06 | 0.811 |
|  |  | T/A | 193 (52.16%) | 213 (46.10%) | 1.355 (0.9676–1.896) | 0.0770 |  | 55 (44.71%) | 27 (39.71%) | 1.372 (0.6605–2.851) | 0.3963 |  | 0.01 | 0.976 |
|  |  | T/T | 98 (26.49%) | 152 (32.90%) | 1 |  |  | 39 (31.71%) | 25 (36.76%) | 1 |  |  |  |  |
|  | Dominant | A/A,T/A | 272 (73.51%) | 310 (69.10%) | 1.294 (0.9422–1.776) | 0.1114 |  | 84 (68.29%) | 43 (63.24%) | 1.241 (0.6382–2.413) | 0.5246 |  | 0.01 | 0.911 |
|  |  | T/T | 98 (26.49%) | 152 (32.90%) | 1 |  |  | 39 (31.71%) | 25 (36.76%) | 1 |  |  |  |  |
|  | Recessive | A/A | 79 (21.35%) | 97 (21.00%) | 0.962 (0.6756–1.369) | 0.8286 |  | 29 (23.58%) | 16 (23.53%) | 0.8728 (0.4124–1.847) | 0.722 |  | 0.05 | 0.819 |
|  |  | T/T,T/A | 291 (78.65%) | 365 (79.00%) | 1 |  |  | 94 (76.42%) | 52 (76.47%) | 1 |  |  |  |  |
|  | Allele | A | 351 (47.43%) | 407 (44.05%) | 1.1 (0.8962–1.349) | 0.3631 |  | 113 (45.93%) | 59 (43.38%) | 1.051 (0.6694–1.651) | 0.8285 |  | 0.03 | 0.857 |
|  |  | T | 389 (52.57%) | 517 (55.95%) | 1 |  |  | 133 (54.07%) | 77 (56.62%) | 1 |  |  |  |  |
|  | Additive | A/A | 79 (21.35%) | 97 (21.00%) | 1.098 (0.8962–1.346) | 0.3662 |  | 29 (23.58%) | 16 (23.53%) | 1.045 (0.6835–1.598) | 0.8385 |  | 0.03 |  |
|  |  | T/A | 193 (52.16%) | 213 (46.10%) |  |  |  | 55 (44.71%) | 27 (39.71%) |  |  |  |  | 0.857 |
|  |  | T/T | 98 (26.49%) | 152 (32.90%) |  |  |  | 39 (31.71%) | 25 (36.76%) |  |  |  |  |  |

^a^Logistic regression: Corrected for sex, age, and BMI, P < 0.05 denotes significance.

**Table S8. Analysis of genotypes for rs12449210 of *HYDIN* and rs777591 of *USP34* in pulmonary function**

| **SNV** | **Model** | **Genotype** | **FEV1%** | | **OR(95%CI)^a^** | **P^a^** |
| --- | --- | --- | --- | --- | --- | --- |
|  |  |  | **≥ 50%** | **< 50%** |  |  |
| rs12449210 of *HYDIN* | Genotype | A/A | 87 (21.22%) | 28 (22.76%) | 1.00 (0.57-1.75) | 0.62 |
|  |  | T/A | 212 (51.71%) | 58 (47.15%) | 1.23 (0.76-1.97) | 0.28 |
|  |  | T/T | 111 (27.07%) | 37 (30.08%) | 1 |  |
|  | Dominant | T/A,A/A | 299 (72.93%) | 86 (69.91%) | 1.15 (0.74-1.79) | 0.54 |
|  |  | T/T | 111 (27.07%) | 37 (30.08%) | 1 |  |
|  | Recessive | A/A | 87 (21.22%) | 28 (22.76%) | 0.88 (0.54-1.42) | 0.6 |
|  |  | T/T,T/A | 323 (78.78%) | 95 (77.24%) | 1 |  |
|  | Allele | A | 386 (47.07%) | 114 (46.34%) | 1.02 (0.77-1.35) | 0.91 |
|  |  | T | 434 (52.93%) | 132 (53.66%) | 1 |  |
|  | Additive | A/A | 87 (21.22%) | 28 (22.76%) | 1.01 (0.76-1.35) | 0.94 |
|  |  | T/A | 212 (51.71%) | 58 (47.15%) |  |  |
|  |  | T/T | 111 (27.07%) | 37 (30.08%) |  |  |
| rs777591 of *USP34* | Genotype | A/A | 37 (9.00%) | 14 (11.29%) | 0.64 (0.32-1.30) | 0.28 |
|  |  | A/G | 186 (45.26%) | 62 (50.00%) | 0.74 (0.48-1.14) | 0.09 |
|  |  | G/G | 188 (45.74%) | 48 (38.71%) | 1 |  |
|  | Dominant | A/G,A/A | 223 (54.26%) | 76 (61.29%) | 0.72 (0.48-1.10) | 0.12 |
|  |  | G/G | 188 (45.74%) | 48 (38.71%) | 1 |  |
|  | Recessive | A/A | 37 (9.00%) | 14 (11.29%) | 0.76 (0.39-1.46) | 0.41 |
|  |  | G/G,A/G | 374 (91.00%) | 110 (88.71%) | 1 |  |
|  | Allele | A | 260 (31.63%) | 90 (36.29%) | 0.81 (0.60-1.09) | 0.17 |
|  |  | G | 562 (68.37%) | 158 (63.71%) | 1 |  |
|  | Additive | A/A | 37 (9.00%) | 14 (11.29%) | 0.78 (0.57-1.07) | 0.12 |
|  |  | A/G | 186 (45.26%) | 62 (50.00%) |  |  |
|  |  | G/G | 188 (45.74%) | 48 (38.71%) |  |  |

^a^Logistic regression: Corrected for sex, age, smoking status, and BMI, P<0.05 denotes significance.

**3. Supplementary Figures**


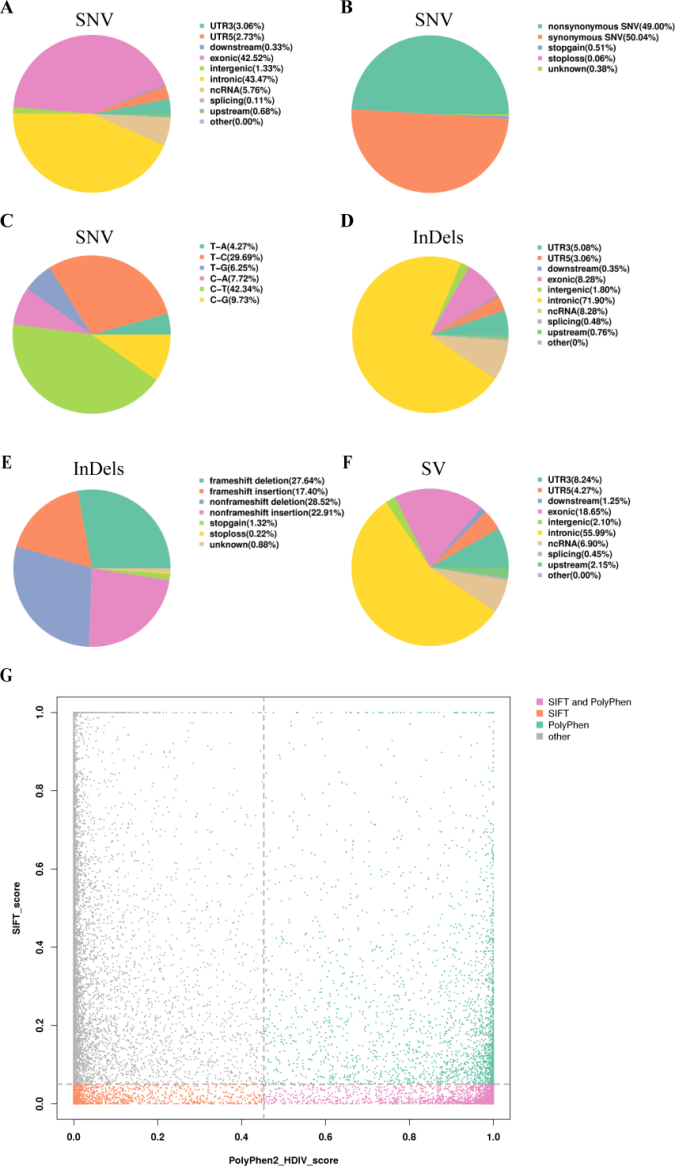


**Supplementary Figure S1. SNVs , InDels, SVs statistics of all samples. (A)** Distribution of SNVs in different regions. **(B)** Distribution of SNVs in exon. **(C)** SNVs base spectrum distribution. **(D)** Distribution of small InDels in different regions. **(E)** Distribution of small InDels in exon. **(F)** Distribution of SVs in different regions. **(G)** SNVs pathogenicity prediction analysis. The abscissa represents Polyphen_HDIV scores, the ordinate represents the SIFT scores. The SIFT scores less than 0.05 were harmful variants, and PolyPhen2_HDIV scores more than 0.453 were harmful variants. The green area means SNVs predicted by Polyphen as harmful, the orange area means SNVs predicted by SIFT as harmful, the pink area means SNVs predicted to be harmful by both two methods, and the gray area represents others.


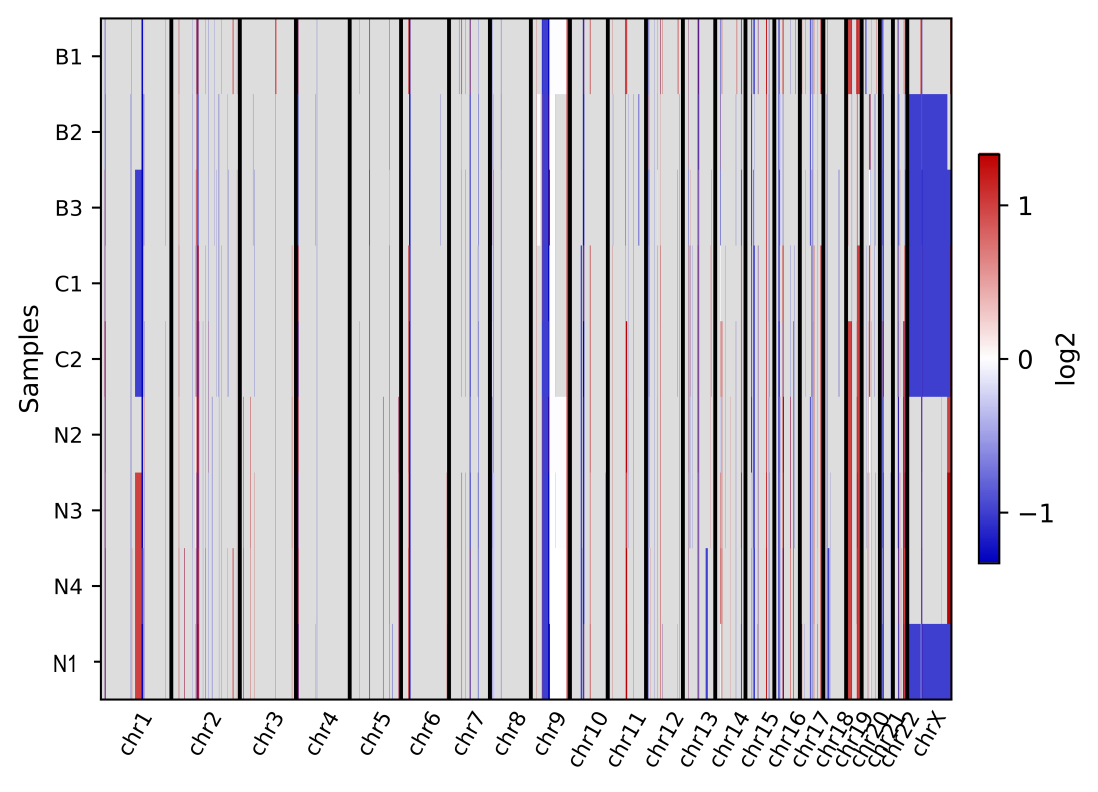


**Supplementary Figure S2. Heat map for CNVs of COPD family subjects.** The abscissa represents the chromosome, the ordinate represents the sample number. Blue = loss, Red = gain.
